# Habitat heterogeneity and food availability in beaver‐engineered streams foster bat richness, activity and feeding

**DOI:** 10.1111/1365-2656.70136

**Published:** 2025-09-15

**Authors:** Valentin Moser, Leonardo Capitani, Luca Zehnder, Alex Hürbin, Martin K. Obrist, Klaus Ecker, Steffen Boch, Silvan Minnig, Christof Angst, Francesco Pomati, Anita C. Risch

**Affiliations:** ^1^ Community Ecology Swiss Federal Institute for Forest, Snow and Landscape Research WSL Birmensdorf Switzerland; ^2^ Department of Aquatic Ecology Eawag: Swiss Federal Institute of Aquatic Science and Technology Dübendorf Switzerland; ^3^ Biodiversity and Conservation Biology Swiss Federal Institute for Forest, Snow and Landscape Research WSL Birmensdorf Switzerland; ^4^ umweltbildner.ch Bern Switzerland; ^5^ info fauna—Nationale Biberfachstelle Neuchâtel Switzerland

**Keywords:** aquatic‐terrestrial, *Castor*, deadwood, ecosystem engineer, feeding guilds, habitat heterogeneity, *Pipistrellus*, structural equation modelling

## Abstract

1. As ecosystem engineers, Eurasian beavers (*Castor fiber*) modify aquatic and terrestrial ecosystems, which can benefit the biodiversity and community composition of plant and animal species. However, in contrast to aquatic taxa, beaver engineering impacts on terrestrial taxa, like bats, are so far largely overlooked. While it has been shown that bats prefer beaver‐engineered ecosystems, the reason for this choice is poorly understood. We hypothesized that this preference may be associated with beaver‐related changes in habitat characteristics and food availability.
2. To address this knowledge gap, we recorded bat species richness, activity and feeding activity in eight beaver‐engineered ecosystems (pool) with paired control sites without beavers (control) along the same stream in Switzerland. In addition, we collected data on food availability (arthropods) with arthropod flight interception traps and characterized habitat suitability with deadwood volume and vegetation surveys, as well as assessing canopy heterogeneity based on different digital height models.
3. The nighly bat species richness increased from four to five species between control and pool sites. Bat activity increased 1.6 times and bat feeding activity 2.3 times in beaver‐engineered systems compared to controls. These increases in richness and activity were explained by higher volumes of standing deadwood, higher canopy heterogeneity and higher arthropod abundance in beaver systems compared to controls.
4. Overall, the volume of standing deadwood, a critical resource for bat roosting and foraging, had a stronger effect on bat species richness than canopy heterogeneity or arthropod availability. Bat feeding guilds (short‐, mid‐, long‐range echolocators) responded differently to beaver‐engineered habitat changes, with edge‐hunting mid‐range species benefiting the most.
5. Our findings suggest that beaver engineering created structurally diverse habitats that supported a broader range of bat species. By modifying both habitat structure and prey abundance, beaver engineering affected bat activity, richness, and feeding activity directly and indirectly. These changes operated across aquatic–terrestrial boundaries, highlighting the cross‐ecosystem influence and ecological complexity of ecosystem engineering.

## Graphical abstract

Beavers reshape ecosystems in ways that benefit bat richness, activity and feeding activity. We linked increased habitat structures (standing deadwood and canopy heterogeneity) and prey abundance to higher bat activity. Our findings uncover how beavers drive biodiversity across aquatic‐terrestrial boundaries.

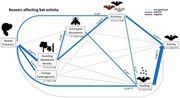

## ZUSAMMENFASSUNG

1. Als Ökosystemingenieure verändern Europäische Biber (*Castor fiber*) aquatische und terrestrische Ökosysteme, was sich positiv auf Biodiversität und Zusammensetzung verschiedener Pflanzen‐ und Tierarten auswirken kann. Im Gegensatz zu aquatischen Arten, wurden die Auswirkungen des Bibers auf terrestrische Arten, wie beispielsweise Fledermäuse, bislang weniger beachtet. Es wurde zwar nachgewiesen, dass Fledermäuse durch den Biber gestaltete Ökosysteme bevorzugen, doch sind die Gründe für diese Präferenz bislang kaum bekannt. Wir stellten die Hypothese auf, dass diese Präferenz der Fledermäuse mit durch den Biber verursachten Veränderungen der Lebensraumeigenschaften und der Nahrungsverfügbarkeit zusammenhängen könnte.
2. Um diese Wissenslücke zu schliessen, haben wir die Artenvielfalt, Gesamtaktivität und Fressaktivität von Fledermäusen in acht von Bibern geschaffenen und gestalteten Ökosystemen (Biberteich) mit gepaarten Kontrollstandorten ohne Biberaktivität (Kontrolle) entlang desselben Baches in der Schweiz erfasst. Darüber hinaus haben wir Daten zur Nahrungsverfügbarkeit (Arthropoden) mit Flugfallen gesammelt und den Lebensraum über das Totholzvolumen und Vegetationserhebungen, sowie mit digitalen Höhenmodellen der Heterogenität des Baumkronendachs charakterisiert.
3. Die nächtliche Artenvielfalt der Fledermäuse stieg im Vergleich mit der Kontrolle in den Biberteichen von vier auf fünf Arten, die Aktivität war 1,6‐mal höher, ebenso die Fressaktivität 2,3‐mal. Diese Zunahme der Artenvielfalt, Aktivität und Fressaktivität lässt sich durch das grössere Volumen an stehendem Totholz, die grössere Heterogenität des Kronendachs und der Anzahl an Arthropoden in den Biber‐Systemen im Vergleich mit den Kontrollstandorten erklären.
4. Insgesamt hatte die Menge an stehendem Totholz, eine wichtige Ressource für Fledermausquartiere und Nahrungssuche, einen stärkeren Einfluss auf die Artenvielfalt der Fledermäuse als die Heterogenität des Baumkronendachs oder die Verfügbarkeit von Arthropoden. Die Arten der drei verschiedenen Fledermausgilden (Kurz‐, Mittel‐ und Langstrecken‐Echolokatoren) reagierten unterschiedlich auf die durch Biber verursachten Habitatsveränderungen, wobei die Arten der Gilde der Mittelstrecken‐Echolokatoren am stärksten profitierten.
5. Unsere Ergebnisse zeigten, dass die von Bibern geschaffenen Lebensräume strukturell vielfältiger waren und eine grössere Bandbreite an Fledermausarten beherbergten. Durch die Veränderung der Lebensraumstruktur als auch der Beutetiere beeinflusste die vom Biber geschaffene Landschaft sowohl direkt als auch indirekt die Artenvielfalt, Aktivität und die Fressaktivität der Fledermäuse. Diese Veränderungen wirkten über aquatische und terrestrische Grenzen hinweg und verdeutlichen den ökosystemübergreifenden Einfluss und die ökologische Komplexität von Ökosystemingenieuren.

## 1 INTRODUCTION

Ecosystem engineers impact biodiversity across habitats through direct and indirect mechanisms (Prugh & Brashares, 2012; Romero et al., 2015; Strickland et al., 2023). Directly, ecosystem engineers modify or create habitats, increasing habitat heterogeneity and providing additional niches for plant and animal species (Hastings et al., 2007; Jones et al., 1994). Indirectly, ecosystem engineers change the composition of local communities, which can lead to novel interactions among species (Sanders & Frago, 2024). The magnitude of direct and indirect effects depends on the ecosystem, the ecosystem engineer and whether the engineer works across habitat boundaries, for example, between water and land (Hastings et al., 2007).

Beavers (*Castor fiber* and *C. canadensis*) are among the most well known ecosystem engineers working across aquatic‐terrestrial habitats. The Eurasian Beaver, *Castor fiber*, made a remarkable recovery after facing near extinction in much of its former range in the 19th century (Halley et al., 2020). As of 2021, an estimated 1.5 million of these ecosystem engineers were re‐transforming landscapes in Eurasia, enhancing aquatic‐terrestrial interactions and habitat heterogeneity, thereby creating more biodiverse environments (Halley et al., 2020; Sommer et al., 2019). Direct effects of beaver engineering, including dam construction and tree felling, enhance biodiversity and promote interactions among animals and plants (Law et al., 2017; Sommer et al., 2019). These primarily positive impacts are well‐documented, especially for aquatic animals like fish and aquatic invertebrates. However, effects of beaver engineering on terrestrial taxa remain less well‐understood (Andersen et al., 2024). Some studies reported direct positive impacts of beavers on reptiles, birds and mammals, but mixed results were found for terrestrial arthropods (Andersen et al., 2024; Fedyń et al., 2023; Wikar et al., 2024). For bats, beavers may indirectly enhance prey availability and food‐web interactions (Ciechanowski et al., 2011; Graham & Goodenough, 2024; Nummi et al., 2011; Orazi et al., 2022). So far, beaver presence has consistently shown to increase bat abundance (Graham & Goodenough, 2024; Hooker et al., 2024; Nummi et al., 2011) and species richness (Ciechanowski et al., 2011; Mori et al., 2025; Orazi et al., 2022), but the underlying mechanisms of these increases remain poorly understood. Two main pathways have been suggested: (1) indirect effects via increased arthropod abundance and changes in arthropod community composition (Ciechanowski et al., 2011; Graham & Goodenough, 2024; Nummi et al., 2011) and (2) direct effects such as improved habitat suitability with increased deadwood volume, more canopy openings and better accessible water areas (Ciechanowski et al., 2011; Menzel et al., 2001). Yet, these pathways remain untested.

Another important research gap is that we know that bat activity increases in beaver‐engineered ecosystems, but we do not know whether feeding activity, a direct indicator of actual foraging, also increases. Further, bat species might also not react uniformly to beaver‐engineered changes, that is, the three different bat feeding guilds, short‐range (SRE), mid‐range (MRE) and long‐range (LRE) echolocation species (Frey‐Ehrenbold et al., 2013). Species of the SRE guild, such as *Myotis* ssp., could profit from increased vegetation complexity in beaver systems. The MRE species, such as *Pipistrellus* spp., prefer hunting along wooded edges (Dietz et al., 2007), a habitat that might increase with beaver engineering. Finally, LRE species, such as *Nyctalus* spp., could profit from a more open canopy in beaver‐engineered systems. As many bat species are declining across Europe, mainly due to habitat loss (Ramírez‐Fráncel et al., 2022), understanding the interactions between bats and beavers could provide valuable insights for bat conservation.

Here, we assessed the effects of beaver engineering on bat species richness, bat activity and bat feeding activity in eight stream ecosystems ranging from near‐natural to heavily human‐impacted, located across the Swiss midlands. We studied how beavers affected bats directly (e.g. through modifications of the habitat structure such as volume of standing deadwood or canopy heterogeneity) and indirectly (e.g. via altered food availability). Specifically, we asked the following three main questions:

Do beaver‐engineered ecosystems support higher bat species richness, activity and feeding activity compared to control sites without beavers?

Are bat species richness, activity and feeding activity associated with differences in standing deadwood volume, canopy heterogeneity and/or arthropod abundance (food availability)?

Do different bat feeding guilds respond differently to beaver‐engineered habitat or food availability changes?

We hypothesized that beaver‐engineered ecosystems would support higher bat richness, activity and feeding activity than control sites (Q1), based on evidence that beavers increase both roosting (e.g. standing deadwood; Larsen et al., 2021; Menzel et al., 2001) and foraging resources (e.g. arthropod abundance; Nummi et al., 2011). For Q2, we expected that arthropod abundance is the strongest predictor of bat activity and feeding activity, as bats concentrate in prey‐rich habitats (Müller et al., 2012; Roeleke et al., 2020). We also expected higher deadwood volume and canopy heterogeneity to support higher bat richness and potentially activity and feeding activity (Hendel et al., 2023; Jung et al., 2012; Tillon et al., 2016). For Q3, we expected MRE and LRE to benefit more from the more open and heterogeneous habitat conditions created by beaver engineering than SRE species (Dietz et al., 2007; Mendes et al., 2017).

## 2 MATERIALS AND METHODS

### 2.1 Study area and species

**FIGURE 1:**
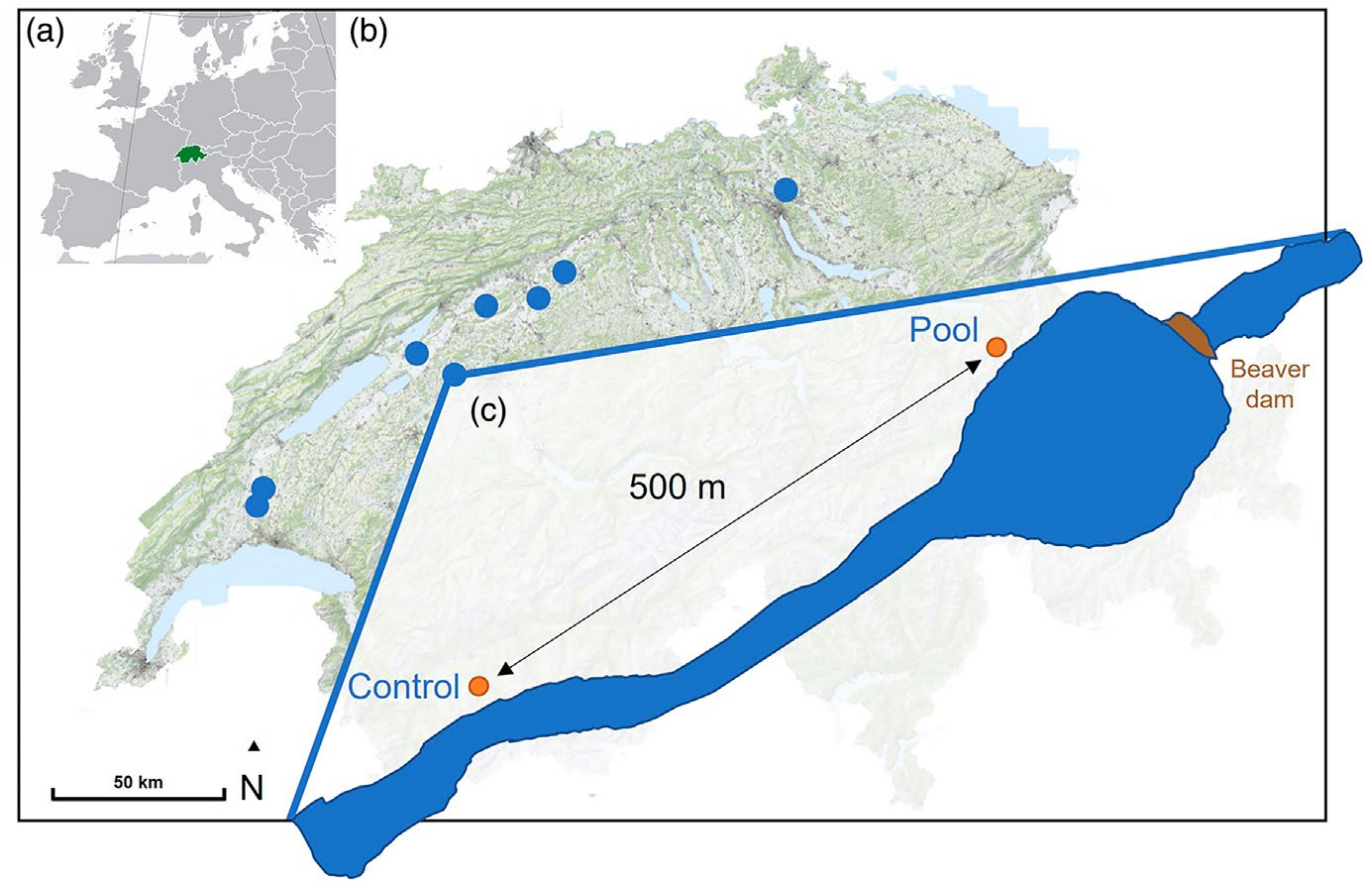
(a) Map of Europe with Switzerland marked in green in the centre. (b) Map of Switzerland showing the eight stream ecosystems (sites) in Switzerland. (c) Schematic of the study design: at each site, we collected data within a beaver‐engineered area with a dam with a beaver pool (Pool) and a control area approximately 500 meters up or downstream in a comparable habitat without beaver engineering (Control). Background ©Swisstopo, Wikimedia commons.

The research was conducted in eight stream ecosystems distributed across the Swiss lowlands (Figure 1, Table S1). We selected an area with an active beaver pool with a dam and pooling water body upstream (hereafter Pool) and a control area located 500 m upstream or downstream from the beaver pool with no or negligible beaver influence (hereafter Control) in each stream ecosystem (Figure 1). For most bats, 500 m is a short flight distance (Lookingbill et al., 2010; Nicholls & Racey, 2006). Thus, if we observe a difference in bat richness, activity and feeding activity between Pool and Control, these changes can be attributed to modifications introduced by beavers rather than to broader landscape effects. Our eight stream ecosystems represented a diversity of typical beaver habitats for Switzerland, located in urban, agricultural and forested areas (Table S1). The chosen streams had comparable Controls to the respective Pools; for example, artificial stream beds in both the Control and Pool if a river was regulated. All relevant cantonal authorities, landowners and governmental agencies were contacted by telephone to obtain permission for access and sampling. Written permission was only necessary for the Talent site. No animal ethics approval was necessary for the work conducted.

### 2.2 Bat data collection and bat call classification

We followed a standard protocol (Figure S1) to collect bat data during the summer of 2022. The recording was conducted using one BatLogger M (detector) each in the Pool and Control area equipped with FG black ultrasonic microphones (FG: an omni directional electric econdensator microphone with integrated preamplifier, highly weather resistant with an integrated temperature sensor, Elekon AG, Lucerne, Switzerland, Figure S1). These omnidirectional microphones have a sensitive range of 10–150 kHz. The microphones were positioned on plastic poles 1.5 m above ground, slightly angled downwards to prevent rain from affecting the sensors. The devices were in place for two times four consecutive recording nights, with a 1‐day break to recharge batteries and extract data from the bat detectors. Hence, we collected bat calls during eight full nights in mid‐June and eight full nights in late July, resulting in 256 record nights (8 nights × 2 month × 8 sites × 2 microphones per site—Pool and Control). Due to full memory cards, empty batteries, and software glitches, records were missing or unusable for 9 of these 256 nights. Fortunately, these nights were not consecutive at any site or period.

The detectors were programmed to record from 15 min before sunset until 15 min after sunrise and recorded continuously with a pre‐trigger of 500 ms and a post‐trigger of 1000 ms, allowing us to capture entire call sequences of passing bats even when the first calls were too faint for the trigger threshold. For each bat taxon, we recorded bat activity (total number of detections) and bat feeding activity (number of detections of feeding buzzes) based on the number of 5‐min intervals in which the taxon was detected. This approach helps mitigate potential biases caused by a bat circling in front of a bat detector, leading to many sequences within a short time window (Britzke et al., 2013). Detected taxa were assigned to the corresponding feeding guild and for each identified bat species, we added the red‐list status of each bat species in Switzerland (Table 2). Many Swiss bat species are endangered (Bohnenstengel et al., 2011) and beaver engineering could potentially help bat conservation. Due to the few missing and incomplete nights, as well as differing night lengths, we standardized the data per night with a factor *α*n *= μ*r/rn, where rn is the recorded time per night at a given site and *μ*r the mean of all recorded times per night across all sites. The recorded bat call sequences were classified using BatScope 4.2.0, trained with a Swiss data set (Obrist & Boesch, 2018). Two algorithms were used to classify bat call sequences: an R‐based Weighted k‐nearest neighbour classifier (KKNN) and a BatScope internal Support Vector Machine (SVM) classifier. The first classifier performs a k‐nearest neighbour classification of a test set using a training set, while the second does a supervised learning algorithm used in machine learning to solve classification and regression tasks. The results of both classifiers were used during manual verification with a special focus on sequences where the classifiers did not agree on identification. First, for the classification, the sequences were automatically cut into calls and then features were extracted and classified at the species level by the two algorithms. Second, filters were applied to exclude insect calls and general noise like call artefacts and microphone bleeps. Finally, the auto‐classified sequences were manually examined with the help of available literature on bats' echolocation calls (Barataud, 2020; Bayerisches Landesamt für Umwelt, 2020; Obrist et al., 2004; Russ, 2021; Zingg, 1990), and social calls (Pfalzer & Kusch, 2003). Sequences with more than one bat species were treated for each species separately. Unambiguous sequences with distinctive call and sequence features were filtered out from further manual checks. The proportion of manually checked sequences depended on the species' rarity and the total number of auto‐classified sequences per species. In case of uncertainty in species determination, sequences were manually verified to the lowest taxonomic level possible. After species verification, a filter to detect bat feeding buzzes, that is, feeding activity (Griffin et al., 1960) was applied and the results were manually checked.

#### 2.2.1 Bat feeding‐guild classification

Sequences were assigned to three species guilds based on their typical foraging behaviour: short‐range echolocators (SRE; *Myotis* spp., *Plecotus* spp., *Barbastella barbastellus*), mid‐range echolocators (MRE; *Hypsugo savii, Miniopterus schreibersii, Pipistrellus* spp.) and long‐range echolocators (LRE; *Eptesicus* spp., *Nyctalus* spp., *Tadarida teniotis, Vespertilio murinus*) (Frey‐Ehrenbold et al., 2013; Froidevaux et al., 2016). Species with calls of bandwidths <30 kHz and durations exceeding 9 ms were classified as LRE. Species with bandwidths >50 kHz and call durations ≤6 ms were assigned to SRE. The remaining species were assigned to MRE, with two exceptions assigned to the SRE: *Plecotus* ssp. with known faint calls and, therefore, a relatively short perception range (Waters & Jones, 1995) and *Barbastella barbastellus*, known to forage around edges and gaps with call intervals of about 90 ms, which hints at a range of approximately 15 m (Denzinger et al., 2001).

#### 2.2.2 Flying arthropod collection and processing

To assess flying arthropod abundance, that is, food availability, we installed flight interception traps (Gossner et al., 2014) as low above the water as possible in the middle of the respective stream body (Pool and Control) at the same times as bat recording (Figure S2). Each trap was constructed of crossed fabrics, measuring 50 cm × 50 cm, with a funnel and collection containers placed at the top and bottom. The containers were filled with 96% technical ethanol. Traps were emptied at the end of each 9‐day sampling period (June and July), and the contents were stored in plastic containers and refrigerated at 4°C until processing. Arthropod abundance was defined as the number of all individuals collected during the two sampling periods in Pool and Control, respectively.

#### 2.2.3 Collection of habitat characteristics data

For the beaver sites, we recorded the age (years of presence of beaver dam) and beaver pond size (greatest width in m). We surveyed all standing and laying (but not submerged) deadwood along a 100‐m long transect each in Pool and Control, extending 10 m from the water edge into the land on both sides of the stream and including everything above water level. In the Pool area, the transect started 25 m below the beaver dam and ended 75 m upstream from the dam. In the Control area, the 100‐m transect was centred on the bat recording location. We calculated the total standing and laying deadwood volume (m^3^) according to Robin and Brang (2009) and the density of deadwood by dividing standing deadwood by transect area (m^3^/m^2^). We also conducted a vegetation survey in a 5 m × 1 m plot centred at the bat recording location. The plot was established parallel to and 1 m away from the water edge. We recorded the total cover of all vascular plant species in the plot (including shrubs and trees). Additionally, vegetation cover was recorded separately per stratum, namely for the herbaceous (<0.5 m), shrub (0.5–3 m) and tree layer (>3 m).

We calculated ‘structure ruggedness’, ‘vegetation ruggedness’ and tree canopy cover based on high‐resolution digital models of 1 m^2^, with values averaged in radii of 100 m around the bat recording locations. These two first variables are known to be useful to model the activity of two bat species (*Rhinolophus hipposideros* and *Myotis myotis*) (Fuchs, 2021). The two height models used (nDSM = normalized digital surface model for structure ruggedness and CHM = canopy height model for canopy ruggedness) were computed from stereo‐matched spectral imagery captured by a Leica ADS sensor, normalized by eliminating terrain height and differed only in the additional extraction of buildings for the canopy height model with a spatial resolution of 1 meter (Ginzler & Hobi, 2016). ‘Structure ruggedness’ and ‘canopy ruggedness’ (further referred to as canopy heterogeneity) were then defined as the mean ruggedness of the total vertical structure >3 m and the mean ruggedness of the canopy >3 m. However, ‘structure ruggedness’ was calculated with the vector ruggedness method VRM (Sappington et al., 2007), while ‘canopy heterogeneity’ was calculated using the curvature function CUR from ArcGIS Pro. Finally, we used the vegetation height model CHM to calculate tree canopy cover (further referred to as ‘canopy’), which is the percentage of the vegetation with a height >3 m.

### 2.3 Data analysis

Statistical analyses were conducted in R version 4.3.1 (R Core Team, 2024). Parts of the data analyses workflows were supported by the language model ChatGPT (OpenAI, versions 3 and 4, accessed in 2024 and 2025). All code and data are available at envidat (Moser et al., 2025). Response variables were checked for collinearity using Spearman‐ranked corrplot (Figure S3). Many of the covariables related to habitat heterogeneity were highly correlated. We therefore included only variables with correlation *p* < |0.5| in our data analyses. To avoid model overfitting given the limited number of sites (*n* = 8), we excluded additional covariates such as pool age, pool width (pool size), and surrounding anthropogenic land use and focused instead on key predictors related to habitat structure and food availability in line with our primary hypotheses. However, we tested the individual effects of pool age (Table S3) and pool size (Table S4) on bat richness, activity and feeding activity in separate generalized linear mixed effects models (GLMMs). Some models did not converge (Tables S3 and S4), and results should therefore be interpreted with caution.

To test the effects of beaver engineering on bat species richness, bat activity, and bat feeding activity, we also used GLMMs with bat species richness, activity and feeding activity as response variables and binomial beaver presence or absence as predictors. Collection date and site were included as random factors to represent the spatial and temporal structure of the study design. These models were built with the package lme4, Version 1.1.34 (Bates et al., 2015). Trigamma *r* ^2^ was calculated with the package MuMIn, Version 1.48.4 (Bartoń, 2024). We used the most appropriate distribution for our response variables, which was a Poisson model for bat richness (overall, SRE, LRE) and negative binomial models for MRE bat richness, bat activity (all) and bat feeding activity (overall, SRE, MRE). We did not calculate a model for LRE feeding activity as we only found LRE feeding activity in 5 out of 246 nights for this guild. We tested all base models for non‐uniformity in residuals, zero‐inflation and overdispersion using the DHARMa package, Version 0.4.6 (Hartig, 2017). If necessary, we allowed the dispersion parameter to vary between Control and Pool using dispformula = ~sample in glmmTMB, Version 1.1.10 (Brooks et al., 2017). This improved model fit and residual diagnostics in the overall activity model, as well as for MRE activity and MRE richness, and therefore these variance structures were retained. For the SRE models, allowing group‐specific dispersion did not improve model diagnostics or inference. We therefore retained the base model for reasons of parsimony and interpretability. The SRE activity model showed acceptable overall fit and no overdispersion. However, DHARMa indicated statistically significant deviation from uniformity. As alternative variance structures did not improve model performance or interpretation, we retained the base model for parsimony. The SRE richness model exhibited strong zero‐deflation and did not outperform the null model. Although the SRE feeding activity model showed acceptable residual structure and no overdispersion or zero inflation, the Levene test indicated heterogeneity of variance across treatments. As the variance model yielded similar estimates and diagnostics, we proceeded with the base model for interpretability.

To better understand the potential relationships between the effect of beaver engineering, habitat characteristics, food availability (arthropod abundance) and bat richness, activity and feeding activity we conducted Structural Equation Models (SEMs) with the package piecewise SEM (version 2.3.0, Lefcheck, 2016). SEMs can uncover linkages in complex networks, and piecewise SEMs, consisting of separate linear models, are generally more robust for nested data structures and smaller data sets (Lefcheck, 2016). The most parsimonious model with the lowest AIC in our piecewise SEM framework was using linear mixed‐effects models with standardized variables and a random effect for site to account for the hierarchical data structure. Although sampling date was initially included as a second random effect, it was excluded after testing revealed it did not substantially improve model performance and led to singular fits. We tested the hypothesis that beaver engineering directly influences habitat characteristics and indirectly influences food availability. We constructed an a priori model based on existing literature and our expectations (Figure S4; Table 1). The model includes both direct and indirect effects of beaver engineering on bat richness, activity and feeding activity, influencing bat activity via changes in standing deadwood, canopy heterogeneity, arthropod abundance, feeding activity and richness. We fitted one model for all bat species combined and separate models for each feeding guild. Additional pathways accounted for unmeasured effects of beaver engineering, such as altered habitat connectivity or prey composition. Due to the low number of observations, we excluded the feeding activity of LRE from the SEM calculated for the LRE guild.

**TABLE 1:** Pathways considered within our a priori SEM model. Path corresponds to the numbered paths in the a priori SEM in Figure S4.

Path	Possible mechanisms	References
1	Beaver engineering increases standing deadwood	Larsen et al. (2021) and Rosell et al. (2005)
2	Beaver engineering increases canopy heterogeneity	Larsen et al. (2021), Law et al. (2017) and Rosell et al. (2005)
3	Beaver engineering increases flying arthropod abundance	Anderson and Rosemond (2007), McCaffery and Eby (2016) and Nummi et al. (2011)
4	Beaver engineering has other effects on bat species richness that are not described in the model (e.g. temporal stability of food supply)	Ciechanowski (2002) and Dietz et al. (2007)
5	Beaver engineering has other effects on bat activity not described in the model (e.g. creating corridors)	Lookingbill et al. (2010)
6	Beaver engineering has other effects on bat feeding activity not described in the model (e.g. more favourable prey composition)	Nummi et al. (2011) and Twining et al. (2018)
7	Arthropod abundance is correlated to the amount of standing deadwood	Parisi et al. (2019) and Stokland et al. (2012)
8	Arthropod abundance is correlated to canopy heterogeneity	Müller et al. (2018)
9	Deadwood is correlated to bat species richness	Hendel et al. (2023) and Tillon et al. (2016)
10	Deadwood is correlated to bat feeding activity	Tillon et al. (2016)
11	Deadwood is correlated to bat activity	Hendel et al. (2023) and Tillon et al. (2016)
12	Canopy heterogeneity is correlated to bat species richness	Jung et al. (2012), Mendes et al. (2017) and Müller et al. 2012
13	Canopy heterogeneity is correlated to bat feeding activity	Jung et al. (2012) and Müller et al. (2012)
14	Canopy heterogeneity is correlated to bat activity	Jung et al. (2012), Mendes et al. (2017) and Müller et al. (2012)
15	Arthropod abundance is correlated to bat species richness	Fukui et al. (2006) and Müller et al. (2012)
16	Arthropod abundance correlated to bat feeding activity	Fukui et al. (2006) and Müller et al. (2012)
17	Arthropod abundance correlated to bat activity	Fukui et al. (2006) and Müller et al. (2012)
18	Bat species richness is correlated to bat feeding activity area	Andreas et al. (2012) and Müller et al. (2012)
19	Bat species richness is correlated to bat activity, that is, multiple different bat species using the beaver system increase bat activity	Ciechanowski et al. (2011) and Orazi et al., 2022
20	Bat feeding activity is correlated to bat activity	Müller et al. (2012) and Nummi et al. (2011)

## 3 RESULTS

### 3.1 Bat richness, activity and feeding activity

**FIGURE 2:**
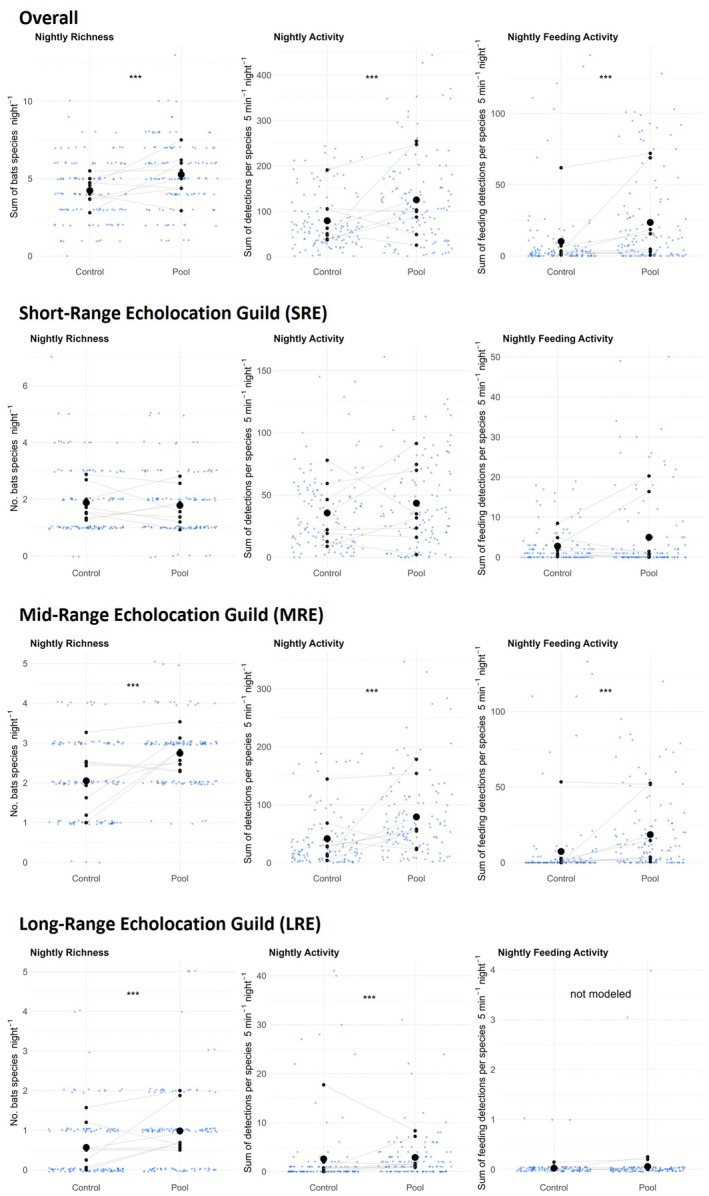
Overall and guild‐specific bat richness (left), bat activity (middle) and bat feeding activity (right) per night (*n* = 16 nights for Control and Pool per site). The small black dots correspond to the means of each site per sampling location (Control and Pool areas) with grey lines connecting each site. The big black dot is the overall mean. For bat richness, the blue dots represent the sum of all identified species per night. For the bat activity and bat feeding activity, the blue dots correspond to the sum of 5‐min activity windows per species. The significance level is indicated with stars: *p < 0.05, **p < 0.01, ***p < 0.001.

We recorded a total of 119,115 bat sequences during the 246 nights across the eight Pool and Control areas. Most of these sequences (80.5%) were assigned to the species level, 19.0% to the single‐genus level and 0.5% to the multi‐genus level (Table 2). In total, we recorded 19 different bat species across all eight sites. The bat species richness in Pool ranged from 9 to 17 species (mean: 12.4 ± SE 0.8) compared to 7 to 12 species (mean: 10.1 ± 0.58) in Control. Per night, we found on average one more bat species in Pool (5.27 ± 0.20) compared to Control areas (4.23 ± 0.17; Figure 2; Table 3, *p* < 0.001). The Pool areas also had more bat species for the MRE (2.74 ± 0.07 vs. 2.05 ± 0.09, *p* < 0.01, Figure 2; Table 3) and LRE guilds (0.98 ± 0.09 vs. 0.57 ± 0.07, *p* < 0.001), but no statistical differences were found between Pool and Control for the SRE guild (1.78 ± 0.10 vs. 1.88 ± 1.11). Overall, we found more Swiss red‐listed bat species (Bohnenstengel et al., 2011) in the Pool than in the Control areas (Table 2).

**TABLE 2:** Level of bat species identification (Level), bat species identifications (Identification), feeding guild (Guild) and red‐list assignment (Red list) based on the raw acoustic recordings.

Level	Identification	Guild		# of sites		Activity			Feeding activity		
			Red list	Control	Pool	Control	Pool	Overall %	Control	Pool	Overall %
Species	*Barbastella barbastellus*	SRE	EN	2	5	25	7	0.03	0	0	0.00
Species	*Myotis bechsteinii*	SRE	VU	7	7	53	115	0.14	0	1	0.01
Species	*Myotis brandtii*	SRE	VU	3	2	46	4	0.04	1	0	0.01
Species	*Myotis daubentonii*	SRE	NT	8	8	699	2900	3.02	0	0	0.00
Species	*Myotis emarginatus*	SRE	EN	7	8	119	50	0.14	2	8	0.07
Species	*Myotis myotis*	SRE	VU	2	4	2	10	0.01	1	0	0.01
Species	*Myotis mystacinus*	SRE	LC	1	1	1	1	0.00	44	279	2.33
Species	*Myotis nattereri*	SRE	NT	7	7	62	54	0.10	0	0	0.00
Species	*Plecotus auritus*	SRE	VU	0	2	0	3	0.00	0	0	0.00
Single‐genus complex	*Myotis* sp.	SRE	—	8	8	10,354	7092	14.65	0	0	0.00
Multi‐genus complex	*Plecotus*/*Myotis* sp.	SRE	—	0	1	0	1	0.00	4	0	0.03
Species	*Hypsugo savii*	MRE	NT	2	2	5	3	0.01	0	4	0.03
Species	*Pipistrellus kuhlii*	MRE	LC	3	6	189	106	0.25	0	4	0.03
Species	*Pipistrellus nathusii*	MRE	LC	6	8	1853	4341	5.20	25	6	0.22
Species	*Pipistrellus pipistrellus*	MRE	LC	8	8	20,508	60,369	67.90	360	256	4.45
Species	*Pipistrellus pygmaeus*	MRE	NT	8	8	146	3386	2.97	4442	6677	80.37
Single‐genus complex	*Pipistrellus* sp.	MRE	—	8	8	1248	3982	4.39	2	606	4.39
Multi‐genus complex	*Hypsugo savii*/*Miniopterus*/*Pipistrellus* sp.	MRE		7	8	221	181	0.34	0	0	0.00
Species	*Eptesicus nilssonii*	LRE	VU	5	7	36	37	0.06	0	0	0.00
Species	*Eptesicus serotinus*	LRE	VU	6	8	318	242	0.47	1	0	0.01
Species	*Nyctalus leisleri*	LRE	NT	2	3	7	16	0.02	344	406	5.42
Species	*Nyctalus noctula*	LRE	NT	1	2	2	15	0.01	1	0	0.01
Species	*Vespertilio murinus*	LRE	VU	3	3	5	18	0.02	178	173	2.54
Single‐genus complex	*Eptesicus* sp.	LRE	—	7	8	133	100	0.20	0	8	0.06
Single‐genus complex	*Nyctalus* sp.	LRE	—	1	2	1	3	0.00	0	0	0.00
Multi‐genus complex	*Eptesicus*/*Nyctalus*/*Vespertilio* sp.	LRE	—	3	5	7	19	0.02	0	0	0.00
Chiroptera not in same guild	—	—	—	2	6	3	17	0.02	0	0	0.00

*Note*: Guilds: LRE, long‐range echolocators; MRE, mid‐range echolocators; SRE, short‐range echolocators. Red list categories: # of sites, number of sites with recordings from each species or species group, split for Pool and Control; —, not applicable; activity, number of sequences for each species or species group in both Control, Pool and its percentage of all sequences (overall %); EN, endangered; feeding activity, feeding buzzes for each species or species group in both Control, Pool and its percentage of all feeding sequences (overall %); LC, least concern; NT, near threatened; VU, vulnerable.

**TABLE 3:** GLMM results for assessing the differences in bat richness, bat activity, and bat feeding activity between Pool and Control overall as well as for the three feeding guilds.

Response variable	Predictor variable	Estimate	SE	*R* ^2^m	*R* ^2^c	*p*‐value
Bat richness	Intercept	1.43	0.07	0.00	0.07	<0.001***
	Pool	0.22	0.06	0.00	0.07	<0.001***
Bat activity	Intercept	4.29	0.18	0.00	0.45	<0.001***
	Pool	0.40	0.10	0.00	0.45	<0.001***
Bat feeding activity	Intercept	1.39	0.44	0.00	0.41	0.002**
	Pool	1.06	0.17	0.00	0.41	<0.001***
SRE bat richness	Intercept	0.60	0.11	0.03	0.11	<0.001***
	Pool	−0.05	0.09	0.03	0.11	0.564
SRE bat activity	Intercept	3.36	0.27	0.03	0.28	<0.001***
	Pool	0.10	0.10	0.03	0.28	0.315
SRE bat feeding activity	Intercept	0.47	0.55	0.13	0.38	0.390
	Pool	−0.20	0.21	0.13	0.38	0.328
MRE bat richness	Intercept	3.64	0.22	0.03	0.11	<0.001***
	Pool	0.55	0.14	0.03	0.11	<0.001***
MRE bat activity	Intercept	3.64	0.22	0.03	0.28	<0.001***
	Pool	0.55	0.14	0.03	0.28	<0.001***
MRE bat feeding activity	Intercept	0.44	0.51	0.13	0.38	0.393
	Pool	1.99	0.25	0.13	0.38	<0.001***
LRE bat richness	Intercept	−0.68	0.21	0.03	0.11	0.001**
	Pool	0.54	0.15	0.03	0.11	<0.001***
LRE bat activity	Intercept	−0.06	0.40	0.03	0.28	0.881
	Pool	0.75	0.20	0.03	0.28	<0.001***
LRE bat feeding activity	Intercept	NA	NA	NA	NA	NA
	Pool	NA	NA	NA	NA	NA

*Note*: LRE feeding activity was not modelled due to insufficient data. Significant relationships (*p* < 0.05) are marked in bold. *p*‐value = indicating significant differences: *p < 0.05, **p < 0.01, ***p < 0.001.

Abbreviations: estimate, estimated value of intercept (equivalent to the Control) and Pool; LRE, long‐ranged echolocation group; MRE, mid‐ranged echolocation group; *R* ^2^c, conditional *R* ^2^, the proportion of variance explained by the fixed and random factors; *R* ^2^m, marginal *R* ^2^, the proportion of variance explained by the fixed factors alone; SE, standard error; SRE, short‐ranged echolocation group.

The overall nightly bat activity per site (Figure 2; Table 3) was 1.6 times higher in the Pool (126.0 ± 8.7) compared to the Control areas (79.7 ± 5.6; *p* < 0.001). We also found higher activity of MRE (79.0 ± 6.16 vs. 41.5 ± 4.49, *p* < 0.001, Figure 2; Table 3) and LRE guild (2.87 ± 0.42 vs. 2.59 ± 0.67, *p* < 0.001) in Pool compared to Control, but no significant difference in activity for the SRE guild (43.5 ± 3.22 vs. 35.6 ± 2.72). Bat feeding activity was found in 16.5% of all recorded sequences. The overall nightly feeding activity (Figure 2; Table 3) was 2.3 times higher in Pool (23.5 ± 2.83) compared to Control (10.2 ± 2.34; *p* < 0.001). For MRE guild (18.5 ± 2.24 vs. 7.39 ± 2.16, *p* < 0.001, Figure 2; Table 3), we found higher feeding activity in the Pool compared to the Control areas, while again, no significant differences were detected for SRE species (4.97 ± 0.90 vs. 2.76 ± 0.40). As mentioned earlier, LRE feeding activity was not modelled due to insufficient data.

### 3.2 Influence of habitat characteristics and arthropod abundance on bat richness, bat activity and bat feeding activity

**FIGURE 3:**
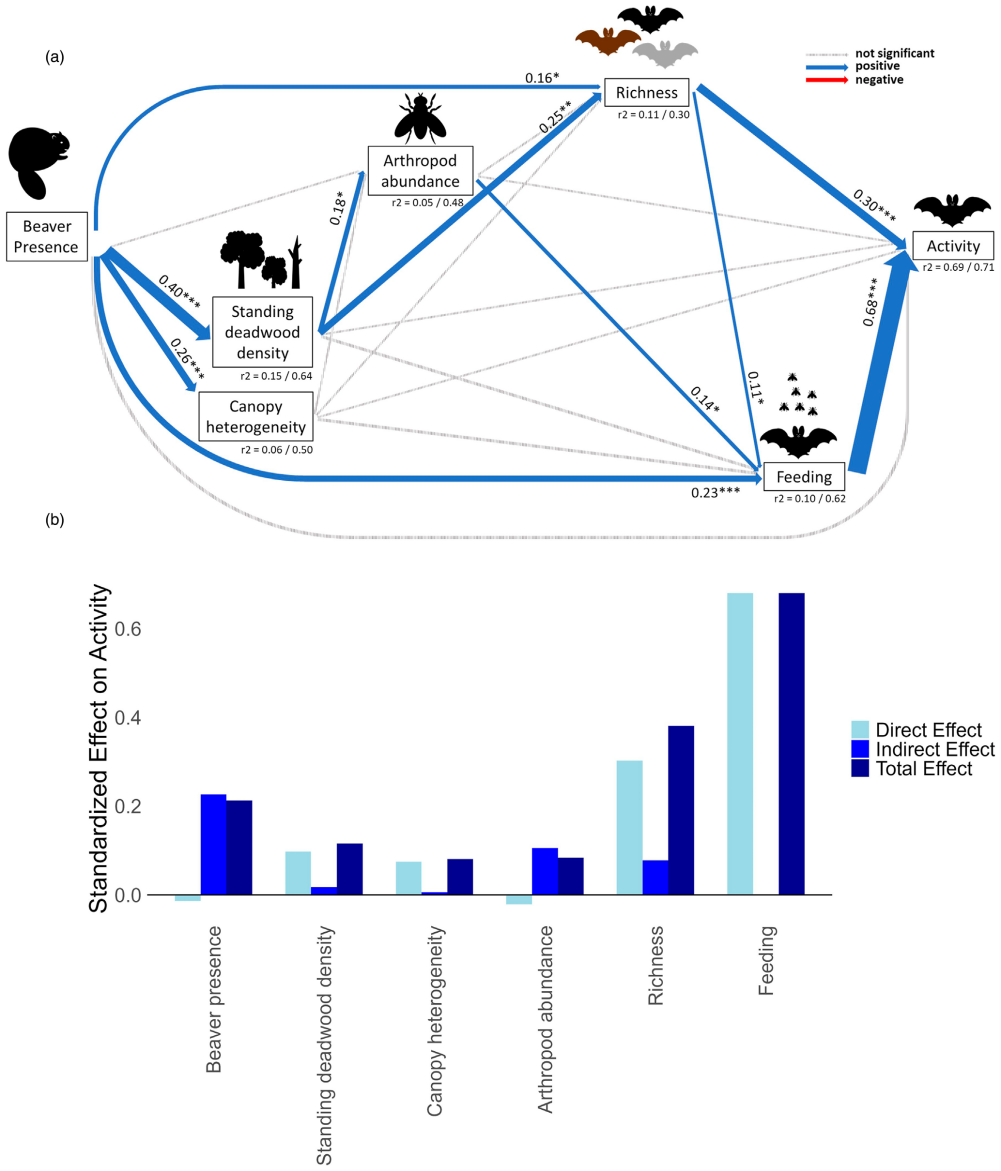
Structural equation model (SEM) testing the correlations between beaver engineering, habitat characteristics, arthropod abundance, bat species richness and bat feeding activity on bat activity. (a) SEM for all bats with icons representing variables included in the model. Thicker solid lines represent significant pathways, with the thickness of the lines corresponding to the standardized estimate, given next to the line with the *p*‐value. Blue arrows represent positive pathways, red arrows represent negative pathways (not found here) and grey‐dashed arrows represent no significant pathways. *p*‐values are indicated as *p < 0.05, **p < 0.01, ***p < 0.001. *r* ^2^ = Marginal *R* ^2^ including only fixed factors and the conditional *R* ^2^ also including random factors. (b) Standardized direct (light blue), indirect (blue) and total effects (dark blue) of all variables on bat activity.

Of the nine variables for which we collected data to describe habitat characteristics and food availability (Table S2), we only retained standing deadwood density, canopy heterogeneity and arthropod abundance due to a high correlation among the variables (see Section 2, Figure S3). The overall SEM for all bats together (Figure 3a; Table S5) revealed a direct and positive relationship between beaver engineering and standing deadwood density, canopy heterogeneity, bat richness and bat feeding activity. Standing deadwood density positively correlated with arthropod abundance and bat species richness. Canopy heterogeneity did not correlate significantly with any of the other variables. Arthropod abundance positively correlated with bat feeding activity and bat richness. Bat activity was positively correlated with both bat richness and bat feeding activity. Overall, we were able to explain 71% of the total variability in bat activity with our model (AIC = 5452.52, Fisher's *C* = 14.33, df = 2, *p* = 0.001), including random factor site (Figure 3a; Table S5), and 69% without random factor site. Overall, the directions of the indirect and total effects (Figure 3b) are similar to the direct effects. The direct effect of beaver engineering and arthropod abundance is negative, but due to positive indirect effects, the total effect of both on bat activity is positive.

**FIGURE 4:**
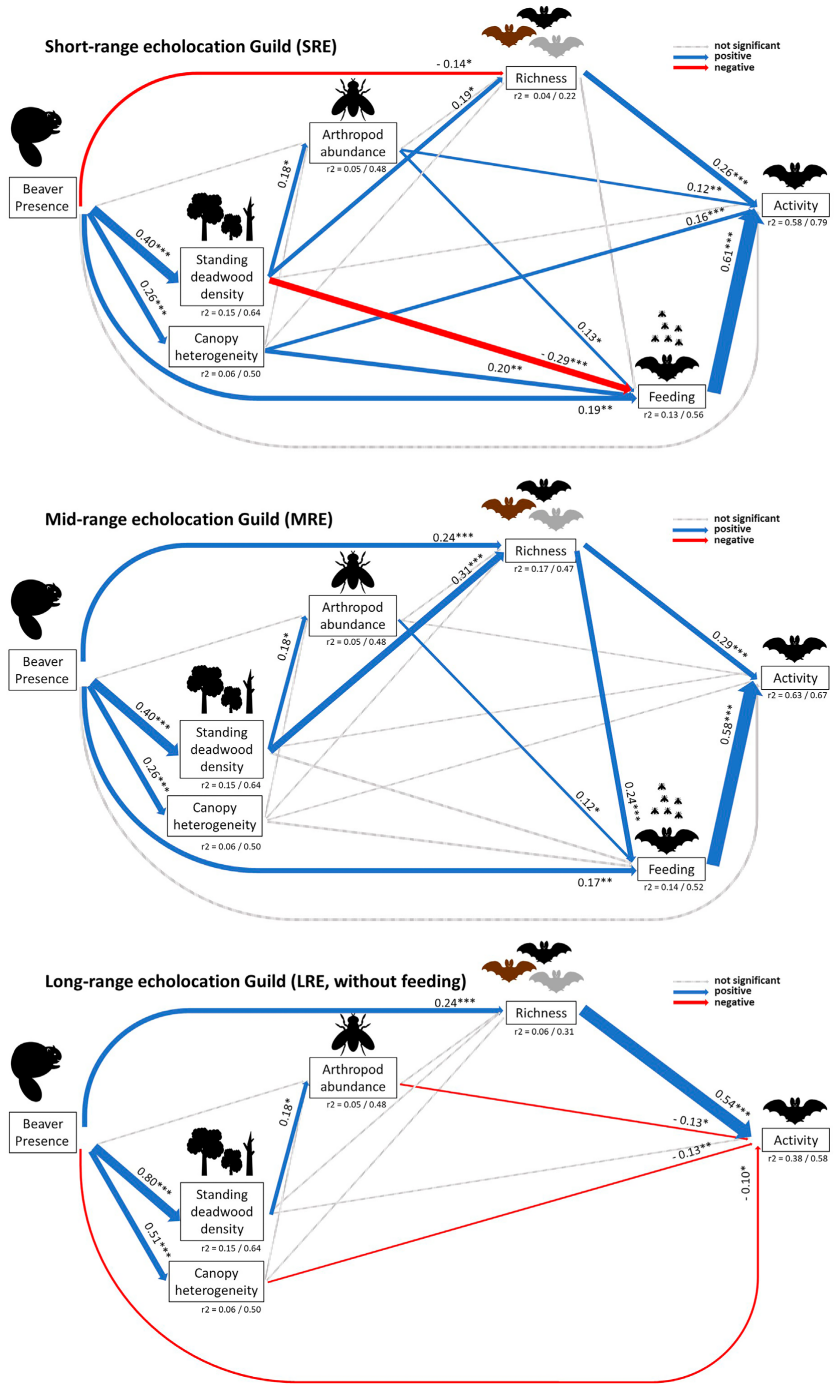
Structural equation models (SEMs) testing the correlations between beaver engineering, habitat characteristics, arthropod abundance, bat species richness and bat feeding activity on bat activity for the three different bat feeding guilds (SRE, MRE, LRE). For LRE, the sample size for feeding activity was not sufficient and therefore it was removed from the model including all relevant paths. The icons represent variables included in the model. Thicker solid lines represent significant pathways, with the thickness of the lines corresponding to the standardized estimate with this number given next to the line with the *p*‐value. Blue arrows represent positive pathways, red arrows represent negative pathways and grey‐dashed arrows represent no significant pathways. *p*‐values are indicated as *p < 0.05, **p < 0.01, ***p < 0.001. *r* ^2^ = Marginal *R* ^2^ including only fixed factors and the conditional *R* ^2^ including also random factors.

We used the same SEM structure for the three different bat guilds, except for LRE, where due to insufficient sample size, we excluded bat feeding activity from the model (Figure 4; Table S5). In all cases, we found a significant positive relationship of beaver engineering on standing deadwood and canopy ruggedness as well as on bat feeding activity for SRE and MRE. Standing deadwood always positively correlated with arthropod abundance. Bat richness was also positively correlated with bat activity for all three guilds and for MRE also with bat feeding activity. For the MRE and LRE guilds, beaver engineering had positive relationships on bat richness (Figure 4). For the MRE and SRE guilds, standing deadwood and bat species richness were positively related. Arthropod abundance was positively related to bat feeding activity for the SRE and MRE guilds and with bat activity for the SRE guild. For the SRE guild, we found significant negative relationships between standing deadwood and bat feeding activity, as well as between beaver presence and bat richness. Further, for the LRE guild, arthropod abundance, canopy ruggedness and beaver presence were negatively related to bat activity (Figure 4). For SRE bat activity, we explained 79% when including the random factor site and 58% without including it (AIC = 4635.95, Fisher's *C* = 14.33, df = 2, *p* = 0.001). For MRE, we explained 67% with random effect and 63% without (AIC =5434.00, Fisher's *C* = 14.33, df = 2, *p* = 0.001), while for LRE the numbers were 58% with versus 38% without random factor (AIC = 2434.12, Fisher's *C* = 14.33, df = 2, *p* = 0.001). Overall, the directions of the total effects were again similar to the direct effects (Figure 4; Figure S5). Exceptions are SRE beaver presence (negative for direct effect to positive for total effect), SRE standing deadwood density (positive to negative), MRE canopy heterogeneity (positive to negative), MRE arthropod abundance (negative to positive) and LRE beaver presence (negative to positive).

## 4 DISCUSSION

Following our expectations, we found that bat species richness, activity and feeding activity were significantly higher in beaver‐engineered Pool compared to Control areas. Our models revealed that this higher bat richness, activity and feeding activity were related to direct beaver engineering effects on standing deadwood density and canopy heterogeneity, as well as indirect effects through arthropod abundance. Notably, and against our expectations, the influence of standing deadwood density and canopy heterogeneity was overall more pronounced than that of arthropod abundance, indicating that habitat characteristics were a better predictor for bat activity than food availability (arthropod abundance). The three different bat feeding guilds differed in their response to beaver engineering, with the edge‐hunting MRE group increasing the most in richness, activity and feeding activity, thus likely driving the overall activity pattern.

### 4.1 Bat richness, bat activity and bat feeding activity

We found large differences in bat richness between the eight sites, with 9–17 different bat species in beaver‐engineered sites. The differences between the sites were likely associated with the fact that our eight sites covered by our sampling design ranged from near‐natural to heavily human‐impacted river systems. For example, artificial light, intensive agricultural production and managed forests (Hendel et al., 2023; Jung et al., 2012; Stone et al., 2009) were shown to reduce bat richness. However, even at sites with high anthropogenic impact, we almost consistently recorded higher bat richness, activity and feeding activity in Pool compared to Control areas. One natural site where we found lower bat activity in the Pool compared to the Control area had duckweed *Lemna* ssp. in the beaver pool. These floating plants are known to impact the echolocation of bats, leading to avoidance behaviour (Ciechanowski et al., 2011). While our eight study sites spanned a gradient from near‐natural to highly anthropogenically influenced environments, we did not include this factor in the main models to avoid overfitting. Nonetheless, we acknowledge that urbanization, forest management and landscape structure may influence local bat communities by altering roost availability or habitat connectivity (Jung et al., 2012; Stone et al., 2009). These factors deserve further investigation in larger‐scale studies. Also, most of our sites were in mid‐successional stages (4–12 years) of beaver presence, and we did not observe any clear effects of pool age on bats (see Table S3). When beaver‐engineered systems continue to age and evolve, it is possible that effects on bats start to deviate from our findings, just like with other animal communities (Bush et al., 2019).

Besides bat richness, previous research also consistently demonstrated higher bat activity in stream sections with beaver presence (Ciechanowski et al., 2011; Graham & Goodenough, 2024; Hooker et al., 2024; Nummi et al., 2011; Orazi et al., 2022). Additionally, we demonstrated an increase in bat feeding activity that was proportionally larger than the increase in activity, providing direct evidence of the importance of beaver systems for bat foraging. We also show how beaver engineering affected the three bat feeding guilds. The higher richness, activity, and feeding activity of the MRE species in Pool compared to Control could be related to increased habitat complexity and higher amounts of open habitat (Mendes et al., 2017). For LRE species, the higher richness and activity in the Pool compared to the Control is also supported by previous results (Hooker et al., 2024; Mori et al., 2025). Although we could not assess LRE feeding activity between Pool and Control due to a low number of detections, we know that LRE bats typically feed higher in the air column than SRE and MRE (Frey‐Ehrenbold et al., 2013) and so may have been out of range of our bat detectors. Alternatively, it is possible that beaver‐engineered ecosystems provide fewer high‐flying insects attractive to LRE bats than we assumed for large water bodies based on the literature (Niga et al., 2023), or that this highly opportunistic bat species guild had their peak feeding activity within our beaver systems outside of our study periods in June and July. We did not find any effects of beaver engineering on SRE richness, activity or feeding activity. However, model diagnostics for this species guild were weaker than for the other two guilds, particularly for richness and abundance, and should therefore be interpreted with caution. Yet, our raw data (Table 2) as well as previous research indicate an increase in the water‐surface foraging species *Myotis daubentonii* in beaver‐engineered ecosystems (Mori et al., 2025; Nummi et al., 2011), which might have balanced declines of other species in this feeding guild. An analysis on species instead of guild level was not done here, but might provide more insights. The mixed responses of our bat feeding guilds to beaver engineering underscore the importance of considering bat species‐specific habitat preferences and foraging strategies when evaluating the ecological impact of beavers on bats.

### 4.2 Habitat heterogeneity and arthropod abundance both contribute to bat richness, activity and feeding activity

#### 4.2.1 Standing deadwood density

Beaver engineering induced flooding often leads to tree mortality due to flooding (Rosell et al., 2005) and therefore to an increase in standing deadwood, which was the environmental variable affecting bat richness most in our models. With bats known to roost in standing deadwood (Tillon et al., 2016), this higher availability of potential roosting places could explain the increased bat richness in Pool compared to Control areas found in our study. Interestingly, we found a strong negative correlation between standing deadwood and bat‐feeding activity for the SRE guild. Standing deadwood in beaver systems is often in flooded areas where no terrestrial vegetation can grow. These flooded areas may be too open for effective hunting by these species, which prefer staying close to vegetation (Frey‐Ehrenbold et al., 2013).

#### 4.2.2 Canopy heterogeneity

Canopy heterogeneity did not have any explanatory power in the overall SEM but was positively correlated with bat activity and bat feeding activity for the SRE species, and negatively with bat activity for the LRE species. SRE bat species were shown to profit from diverse canopy structures, enhancing bat foraging efficiency and habitat use by providing a variety of different hunting niches (Jung et al., 2012; Müller et al., 2012). Also, arthropods profit from heterogeneous canopy cover (Müller et al., 2018). Thus, higher canopy heterogeneity might also stand for better bat feeding grounds. Opposite to SRE species, the LRE species prefer open canopy with few or no trees (Blakey et al., 2017), which explains our negative correlation between LRE species activity and canopy heterogeneity.

#### 4.2.3 Arthropod abundance

Deadwood is a resource for arthropods and supports saproxylic species like beetles, gnats, flies, and moths (Parisi et al., 2019; Stokland et al., 2012). Our SEMs revealed positive relationships between deadwood and arthropod abundance. Arthropod abundance was then positively related to bat feeding activity for MRE and SRE. For the SRE guild, the positive relationship between feeding activity and arthropod abundance could be driven by one species: *Myotis daubentonii* (Table 2). This species preferably hunts low over slow‐flowing watercourses and therefore could profit more from the beaver engineering than other species (Hooker et al., 2024; Mori et al., 2025). In the overall SEM, the most likely explanation for the positive relationship between arthropod abundance and bat feeding activity is that beaver systems attract many different insects (Anderson & Rosemond, 2007; McCaffery & Eby, 2016; Nummi et al., 2011). This high abundance and diversity of arthropods likely also attract bat species specialized in different prey species (Andreas et al., 2012). However, in contrast to our expectations, arthropod abundance had less explanatory power in our models compared to variables that were directly modified by beavers, for example, standing deadwood or canopy heterogeneity. This could be because many of the bat species we recorded prefer familiar hunting areas rather than hunt opportunistically (Hillen et al., 2009; Kerth et al., 2001).

#### 4.2.4 Other potential explanatory variables explaining bat richness, activity and feeding activity

The direct positive and negative links between beaver engineering and bat richness, bat activity and bat feeding activity in our SEMs indicate that with the variables measured in our study we cannot explain all the variability in how beavers influence bats. Other variables are therefore important to explain the impact of beaver engineering on bats. Increased open water area due to beaver engineering (Rosell et al., 2005; Sommer et al., 2019) provides additional drinking areas and larger corridors for hunting and flying (Ciechanowski, 2002; Lookingbill et al., 2010). However, in our study, we did not detect any relationship between pool size and bat richness, activity or feeding except for a positive, significant effect on the feeding activity of SRE species (Table S4). This is likely due to limited variability in measured pond widths and the relatively small number of studied beaver sites. Another important variable, and of special importance to the MRE species (Frey‐Ehrenbold et al., 2013), could be the amount of edge habitat (Fukui et al., 2006; Morris et al., 2010). MRE species showed the most substantial increases in richness and abundance in beaver‐engineered ecosystems compared to the other feeding guilds, also in other studies (Ciechanowski et al., 2011; Orazi et al., 2022). Besides the number of arthropods, the arthropod community composition could also drive the increase in bat feeding activity. Beavers increase emergent insect species with aquatic larval stages (e.g. certain Diptera or Trichoptera, Nummi et al., 2011). This could lead to a higher amount of fatty acids in the available food (Twining et al., 2018). Higher amounts of fatty acids, such as omega‐3s, were shown to be a primary driver of breeding success for riparian birds (Twining et al., 2018) and could also be important for bats.

#### 4.2.5 Beavers as ecosystem engineers could support ecosystem conservation beyond aquatic habitats

Our findings align with the notion that ecosystem engineering in general corresponds with a 25% increase in species richness (Romero et al., 2015). Our results show that beaver engineering‐related changes to habitat structure and food availability directly and indirectly promote higher bat richness, activity and feeding activity. Importantly, this included a higher number of red‐listed bat species, suggesting that beaver‐engineered habitats may offer valuable foraging or roosting resources for species of conservation concern. While the full roles of bats in aquatic‐terrestrial linkages remain unclear, our study adds to the growing evidence that beaver ponds can positively influence biodiversity beyond the aquatic zone (Fedyń et al., 2023; McCaffery & Eby, 2016). In landscapes heavily modified by humans, beavers could therefore serve as natural agents of ecological restoration (Coggan et al., 2018; Law et al., 2017), with the potential to benefit functionally different taxa across aquatic‐terrestrial boundaries.

## AUTHOR CONTRIBUTIONS

Valentin Moser, Silvan Minnig, Christof Angst, Anita C. Risch and Francesco Pomati conceived the ideas and designed the study; Valentin Moser, Luca Zehnder, Alex Hürbin and Steffen Boch contributed to the data collection; Martin K. Obrist verified bat echolocation calls; Klaus Ecker provided a comprehensive set of variables derived from a variety of national GIS databases and remotely sensed data for incorporation into the modelling process; Valentin Moser, Luca Zehnder and Leonardo Capitani analysed the data; Valentin Moser led the writing of the manuscript. Anita C. Risch and Francesco Pomati jointly wrote the proposal for funding, organized and led the overall study. All authors contributed critically to the drafts and gave final approval for publication.

## FUNDING INFORMATION

ETH Board (Grant/Award: Blue‐Green Biodiversity Research Initiative) and BAFU (Grant/Award: Nationales Biberprojekt).

## CONFLICT OF INTEREST STATEMENT

The authors declare no competing interests.

## STATEMENT ON INCLUSION

Our study brings together stakeholders and scientists located in Switzerland, where the study was conducted. Together, we worked from the start to define the research and design the study, which ensured diverse sets of perspectives from the onset. Whenever relevant, literature published by scientists from the region was cited; efforts were made to consider relevant work published in the local language as well. We are also conducting outreach in the form of public presentations.

## Supporting information


**Table S1.** Variables describing the site characteristics of our eight sites. Site: name of each stream; location: indicates if it was a pool area with beaver presence or control; latitude and longitude: coordinates EW and NS; age: time in year since beaver presence in the pool area; habitat: open, more agricultural/urban type of setting or forest setting; pool width: width of the beaver pool in metres for the beaver pool; and control location indicates if the control location was up‐ or downstream of the beaver system.
**Table S2.** Differences between habitat characteristics and arthopod abundances between pool and control. Unit, mean and standard deviation and the *p*‐value with * indicating whether we found significant differences between pool and control areas for these variables with a paired *t*‐test (*p < 0.05, **p < 0.01, ***p < 0.001). Vegetation cover = % cover of all vegetation above a 1 × 5 m plot. Cumulative cover = % cover of the herb, shrub and tree layer added separately. Deadwood volume = laying and standing deadwood (m^3^) above the water. Deadwood density = deadwood volume divided by the survey area. Structure ruggedness and canopy heterogeneity are unitless modelled values based on a digital height models. Arthropods abundance = number caught individuals.
**Table S3.** GLMM results assessing the effect of beaver pool age on bat richness, bat activity and bat feeding activity overall and for the three feeding guilds. Estimate, estimated effect of age; LRE, long‐ranged echolocation group; MRE, mid‐ranged echolocation group; *p*‐value, significance of fixed effects: *p < 0.05, **p < 0.01, ***p < 0.001; *R* ^2^c, conditional *R* ^2^, the proportion of variance explained by fixed and random effects; *R* ^2^m, marginal *R* ^2^, the proportion of variance explained by fixed effects alone; SRE, short‐ranged echolocation group; SE, standard error. Several models showed convergence warnings or boundary (singular) fits, likely due to limited sample size (*n* = 8 sites). For singular fit, this was richness_all, feeding_all, richness_SRE, feeding_SRE, richness_MRE, feeding_MRE, richness_LRE. For gradient convergence failure this was feeding_SRE, abundance_LRE, feeding_LRE. Models should therefore be interpreted with caution.
**Table S4.** GLMM results assessing the effect of beaver pool size (maximum width) on bat richness, bat activity and bat feeding activity overall and for the three feeding guilds. Estimate, estimated effect of pool size; LRE, long‐ranged echolocation group; MRE, mid‐ranged echolocation group; *p*‐value, significance of fixed effects: *p < 0.05, **p < 0.01, ***p < 0.001; *R* ^2^c, conditional *R* ^2^, the proportion of variance explained by fixed and random effects; *R* ^2^m, marginal *R* ^2^, the proportion of variance explained by fixed effects alone; SRE, short‐ranged echolocation group; SE, standard error. Several models showed convergence warnings or boundary (singular) fits, likely due to limited sample size (*N* = 8 sites). For singular fit, this was richness_all, feeding_all, richness_SRE, feeding_SRE, richness_MRE, feeding_MRE, richness_LRE. For gradient convergence failure, this was feeding_SRE, feeding_MRE, abundance_LRE, richness_LRE, feeding_LRE. Models should therefore be interpreted with caution.
**Table S5.** Results of the SEM for all bats (overall) and for each of the three bat feeding guilds. Crit. value, critical value (estimate divided by SE); df, degrees of freedom; estimate, estimated value of predictor effects; *p*‐value, indicates the significance of the predictor effects: *p < 0.05, **p < 0.01, ***p < 0.001; SE, standard error of the estimate; Std. estimate, standardized estimate indicating the effect size in standardized units.
**Figure S1.** Schematic protocol for analysing the bat acoustic data. Autonomous bat recorders were deployed for 2 weeks. Following data collection, the raw data files were processed with Batsscope (Obrist & Boesch, 2018), a program that cuts and classifies bat calls based on machine learning. Experts verified the results. Further data preparation was conducted in R (R Core Team, 2024) by assigning call sequences into 5‐minute intervals. Sources: Batlogger with permission from batlogger.com, R‐Logo from https://www.r‐project.org/logo/Rlogo.svg under CC‐BY‐SA 4.0 licence.
**Figure S2.** Flight interception trap installed over the pool behind the beaver dam in one of the stream ecosystems included in our study. Photo credits: Valentin Moser.
**Figure S3.** Spearman correlations between the habitat variables and abundance of arthropods included in our study. abundance_arthropods = abundance arthropods; canopy = canopy cover (proportion between 0 and 1); canopy_heterogeneity = canopy heterogeneity (unitless modelled value); cumulative_cover = cumulative cover of herb, shrub and tree layer (%); deadwood = deadwood (total deadwood volume in m^3^); deadwood_standing = total standing deadwood (standing deadwood volume in m^3^); deadwood_standing_area = standing deadwood density (standing deadwood volume in m^3^ divided by survey area in m^2^); structure_ruggedness = structure ruggedness (unitless modelled value); vegetation_cover = vegetation cover (%).
**Figure S4.** A priori SEM model. The arrows represent all possible pathways based on our hypothesis. The path numbers correspond to numbers in Table 1, which indicates probable mechanisms and references. All paths are presumed to be positive and going from left to right.

## Data Availability

Data available from the envidat portal at the Swiss Federal Institute for Forest, Snow and Landscape Research WSL, Birmensdorf, Switzerland https://doi.org/10.16904/envidat.659.
