# Supplementary material for: Habitat heterogeneity and food availability in beaver‐engineered streams foster bat richness, activity and feeding
Source: J Anim Ecol. 2025 Sep 15;94(12):2403–20. doi: 10.1111/1365-2656.70136 (PMC12673235; doi:10.1111/1365-2656.70136)
Supplement: Supplementary file 1 — Table S1. Variables describing the site characteristics of our eight sites. Site: name of each stream; location: indicates if it was a pool area with beaver presence or control; latitude and longitude: coordinates EW and NS; age: time in year since beaver presence in the pool area; habitat: open, more agricultural/urban type of setting or forest setting; pool width: width of the beaver pool in metres for the beaver pool; and control location indicates if the control location was up‐ or downstream of the beaver system. Table S2. Differences between habitat characteristics and arthopod abundances between pool and control. Unit, mean and standard deviation and the p‐value with * indicating whether we found significant differences between pool and control areas for these variables with a paired t‐test (*p < 0.05, **p < 0.01, ***p < 0.001). Vegetation cover = % cover of all vegetation above a 1 × 5 m plot. Cumulative cover = % cover of the herb, shrub and tree layer added separately. Deadwood volume = laying and standing deadwood (m3) above the water. Deadwood density = deadwood volume divided by the survey area. Structure ruggedness and canopy heterogeneity are unitless modelled values based on a digital height models. Arthropods abundance = number caught individuals. Table S3. GLMM results assessing the effect of beaver pool age on bat richness, bat activity and bat feeding activity overall and for the three feeding guilds. Estimate, estimated effect of age; LRE, long‐ranged echolocation group; MRE, mid‐ranged echolocation group; p‐value, significance of fixed effects: *p < 0.05, **p < 0.01, ***p < 0.001; R 2c, conditional R 2, the proportion of variance explained by fixed and random effects; R 2m, marginal R 2, the proportion of variance explained by fixed effects alone; SRE, short‐ranged echolocation group; SE, standard error. Several models showed convergence warnings or boundary (singular) fits, likely due to limited sample size (n = 8 sites). For singular fit, thi [file JANE-94-2403-s001.docx]

**SUPPORTING INFORMATION**
**Supplementary material**

**Table S1.** Variables describing the site characteristics of our eight sites. Site: name of each stream; location: indicates if it was a pool area with beaver presence or control; latitude and longitude: coordinates EW and NS; age: time in year since beaver presence in the pool area; habitat: open, more agricultural/urban type of setting or forest setting; pool width: width of the beaver pool in metres for the beaver pool; and control location indicates if the control location was up‐ or downstream of the beaver system.

| **Site** | **Location** | **Latitude** | **Longitude** | **Age (years)** | **Habitat** | **Pool width  (m)** | **Control Location** |
| --- | --- | --- | --- | --- | --- | --- | --- |
| Chriesbach | Control | 47.406719 | 8.6221082 | NA | open, urban | NA | downstream |
| Chriesbach | Pool | 47.408572 | 8.628514 | 4 | open, urban | 8 | downstream |
| Leugene | Control | 47.170763 | 7.3294246 | NA | open, agriculture | NA | upstream |
| Leugene | Pool | 47.169084 | 7.3230668 | 5 | open, agriculture | 5 | upstream |
| Gaebelbach | Control | 46.946011 | 7.3356266 | NA | open, agriculture | NA | downstream |
| Gaebelbach | Pool | 46.946162 | 7.3431418 | 5 | open, agriculture | 7 | downstream |
| Weierbach | Control | 47.16973 | 7.63332 | NA | open, near-natural | NA | upstream |
| Weierbach | Pool | 47.166004 | 7.6257041 | 11 | open, near-natural | 32 | upstream |
| Haslibach | Control | 46.989526 | 7.2240011 | NA | Forest, human-impacted | NA | upstream |
| Haslibach | Pool | 46.994263 | 7.2267517 | 8 | Forest, human-impacted | 8 | upstream |
| Riedgrabe | Control | 47.209973 | 7.7176908 | NA | Forest, human-impacted | NA | downstream |
| Riedgrabe | Pool | 47.204321 | 7.7240172 | 12 | Forest, human-impacted | 27 | downstream |
| Coruz | Control | 46.642441 | 6.6869508 | NA | Forest, near-natural | NA | downstream |
| Coruz | Pool | 46.638383 | 6.6838381 | 11 | Forest, near-natural | 6 | downstream |
| Talent | Control | 46.583501 | 6.6818291 | NA | Forest, near-natural | NA | upstream |
| Talent | Pool | 46.586815 | 6.6816326 | 5 | Forest, near-natural | 12 | upstream |

**Table S2.** Differences between habitat characteristics and arthropod abundances between Pool and Control. Unit, mean and standard deviation and the p‐value with * indicating whether we found significant differences between pool and control areas for these variables with a paired t‐test (*p < 0.05, **p < 0.01, ***p < 0.001). Vegetation cover = % cover of all vegetation above a 1 × 5 m plot. Cumulative cover = % cover of the herb, shrub and tree layer added separately. Deadwood volume = laying and standing deadwood (m3) above the water. Deadwood density = deadwood volume divided by the survey area. Structure ruggedness and canopy heterogeneity are unitless modelled values based on digital height models. Arthropods abundance = number of caught individuals.

| **Variable** | **Unit** | **Control:  Mean ± SD** | **Pool:  Mean ± SD** | **p_value** |  |
| --- | --- | --- | --- | --- | --- |
| vegetation cover | % [0-100] | 95.90 ± 4.87 | 85.80 ± 10.15 | 0.04 | * |
| cumulative cover | % percent [0-300] | 112.94 ± 28.86 | 101.27 ± 26.39 | 0.49 |  |
| deadwood | Volume [m^3^] | 8.07 ± 11.13 | 40.95 ± 30.88 | 0.02 | * |
| standing deadwood total | Volume [m^3^] | 2.28 ± 3.59 | 17.05 ± 20.62 | 0.11 |  |
| standing deadwood density | Density [volume m^3^ per m^2^] | 0.0010 ± 0.0017 | 0.0054 ± 0.0067 | 0.13 |  |
| structure ruggedness | unitless | 0.06 ± 0.05 | 0.08 ± 0.03 | 0.19 |  |
| canopy heterogeneity | unitless | -0.07 ± 0.18 | 0.01 ± 0.14 | 0.36 |  |
| canopy cover | proportion [0-1] | 0.41 ± 0.34 | 0.64 ± 0.24 | 0.09 |  |
| abundance arthropods | number [n] | 176.09 ± 110.07 | 209.05 ± 96.23 | 0.13 |  |

**Table S3.** GLMM results assessing the effect of beaver pool age on bat richness, bat activity and bat feeding activity overall and for the three feeding guilds. Estimate, estimated effect of age; LRE, long‐ranged echolocation group; MRE, mid‐ranged echolocation group; p‐value, significance of fixed effects: *p < 0.05, **p < 0.01, ***p < 0.001; R2c, conditional R2, the proportion of variance explained by fixed and random effects; R2m, marginal R2, the proportion of variance explained by fixed effects alone; SRE, short‐ranged echolocation group; SE, standard error. Several models showed convergence warnings or boundary (singular) fits, likely due to limited sample size (n = 8 sites). For singular fit, this was richness_all, feeding_all, richness_SRE, feeding_SRE, richness_MRE, feeding_MRE, richness_LRE. For gradient convergence failure this was feeding_SRE, abundance_LRE, feeding_LRE. Models should therefore be interpreted with caution.

| **Model** | **Term** | **Estimate** | **Std. Error** | **R2m** | **R2c** | **p-value** | **Significance** |
| --- | --- | --- | --- | --- | --- | --- | --- |
| abundance_all | (Intercept) | 4.70 | 0.68 | 0.00 | 0.56 | 0.00 | *** |
| abundance_all | age | -0.02 | 0.08 | 0.00 | 0.56 | 0.84 |  |
| richness_all | (Intercept) | 1.35 | 0.22 | 0.05 | 0.20 | 0.00 | *** |
| richness_all | age | 0.04 | 0.03 | 0.05 | 0.20 | 0.15 |  |
| feeding_all | (Intercept) | 2.38 | 1.44 | 0.00 | 0.73 | 0.10 |  |
| feeding_all | age | -0.02 | 0.17 | 0.00 | 0.73 | 0.90 |  |
| abundance_SRE | (Intercept) | 3.08 | 1.05 | 0.01 | 0.71 | 0.00 | ** |
| abundance_SRE | age | 0.03 | 0.13 | 0.01 | 0.71 | 0.79 |  |
| richness_SRE | (Intercept) | 0.12 | 0.28 | 0.03 | 0.09 | 0.67 |  |
| richness_SRE | age | 0.05 | 0.03 | 0.03 | 0.09 | 0.10 |  |
| feeding_SRE | (Intercept) | 0.68 | 2.01 | 0.02 | 0.80 | 0.74 |  |
| feeding_SRE | age | -0.11 | 0.24 | 0.02 | 0.80 | 0.65 |  |
| abundance_MRE | (Intercept) | 4.45 | 0.65 | 0.02 | 0.54 | 0.00 | *** |
| abundance_MRE | age | -0.04 | 0.08 | 0.02 | 0.54 | 0.57 |  |
| richness_MRE | (Intercept) | 0.99 | 0.15 | 0.00 | 0.00 | 0.00 | *** |
| richness_MRE | age | 0.00 | 0.02 | 0.00 | 0.00 | 0.89 |  |
| feeding_MRE | (Intercept) | 2.18 | 1.38 | 0.00 | 0.69 | 0.11 |  |
| feeding_MRE | age | -0.02 | 0.17 | 0.00 | 0.69 | 0.92 |  |
| abundance_LRE | (Intercept) | -0.08 | 0.78 | 0.03 | 0.34 | 0.92 |  |
| abundance_LRE | age | 0.09 | 0.09 | 0.03 | 0.34 | 0.34 |  |
| richness_LRE | (Intercept) | -0.59 | 0.48 | 0.02 | 0.11 | 0.22 |  |
| richness_LRE | age | 0.06 | 0.06 | 0.02 | 0.11 | 0.29 |  |
| feeding_LRE | (Intercept) | -27.08 | 8.06 | 0.00 | 1.00 | 0.00 | *** |
| feeding_LRE | age | 0.19 | 0.93 | 0.00 | 1.00 | 0.84 |  |

**Table S4.** GLMM results assessing the effect of beaver pool size (maximum width) on bat richness, bat activity and bat feeding activity overall and for the three feeding guilds. Estimate, estimated effect of pool size; LRE, long‐ranged echolocation group; MRE, mid‐ranged echolocation group; p‐value, significance of fixed effects: *p < 0.05, **p < 0.01, ***p < 0.001; R2c, conditional R2, the proportion of variance explained by fixed and random effects; R2m, marginal R2, the proportion of variance explained by fixed effects alone; SRE, short‐ranged echolocation group; SE, standard error. Several models showed convergence warnings or boundary (singular) fits, likely due to limited sample size (N = 8 sites). For singular fit, this was richness_all, feeding_all, richness_SRE, feeding_SRE, richness_MRE, feeding_MRE, richness_LRE. For gradient convergence failure, this was feeding_SRE, feeding_MRE, abundance_LRE, richness_LRE, feeding_LRE. Models should therefore be interpreted with caution.

| **Model** | **Term** | **Estimate** | **Std. Error** | **R2m** | **R2c** | **p-value** | **Significance** |
| --- | --- | --- | --- | --- | --- | --- | --- |
| abundance_all | (Intercept) | 4.44 | 0.43 | 0.01 | 0.56 | 0.00 | *** |
| abundance_all | pool_size | 0.01 | 0.03 | 0.01 | 0.56 | 0.67 |  |
| richness_all | (Intercept) | 1.54 | 0.15 | 0.02 | 0.20 | 0.00 | *** |
| richness_all | pool_size | 0.01 | 0.01 | 0.02 | 0.20 | 0.45 |  |
| feeding_all | (Intercept) | 1.44 | 0.84 | 0.11 | 0.73 | 0.09 |  |
| feeding_all | pool_size | 0.06 | 0.05 | 0.11 | 0.73 | 0.25 |  |
| abundance_SRE | (Intercept) | 3.20 | 0.66 | 0.01 | 0.71 | 0.00 | *** |
| abundance_SRE | pool_size | 0.01 | 0.04 | 0.01 | 0.71 | 0.80 |  |
| richness_SRE | (Intercept) | 0.48 | 0.21 | 0.00 | 0.10 | 0.02 | * |
| richness_SRE | pool_size | 0.00 | 0.01 | 0.00 | 0.10 | 0.73 |  |
| feeding_SRE | (Intercept) | -0.34 | 0.00 | 0.00 | 0.78 | 0.00 | *** |
| feeding_SRE | pool_size | 0.01 | 0.00 | 0.00 | 0.78 | 0.00 | *** |
| abundance_MRE | (Intercept) | 3.90 | 0.41 | 0.03 | 0.54 | 0.00 | *** |
| abundance_MRE | pool_size | 0.02 | 0.03 | 0.03 | 0.54 | 0.53 |  |
| richness_MRE | (Intercept) | 0.98 | 0.09 | 0.00 | 0.00 | 0.00 | *** |
| richness_MRE | pool_size | 0.00 | 0.01 | 0.00 | 0.00 | 0.67 |  |
| feeding_MRE | (Intercept) | 1.21 | 0.79 | 0.13 | 0.68 | 0.13 |  |
| feeding_MRE | pool_size | 0.06 | 0.05 | 0.13 | 0.68 | 0.18 |  |
| abundance_LRE | (Intercept) | 0.34 | 0.51 | 0.02 | 0.33 | 0.51 |  |
| abundance_LRE | pool_size | 0.02 | 0.03 | 0.02 | 0.33 | 0.51 |  |
| richness_LRE | (Intercept) | -0.25 | 0.32 | 0.00 | 0.08 | 0.43 |  |
| richness_LRE | pool_size | 0.01 | 0.02 | 0.00 | 0.08 | 0.62 |  |
| feeding_LRE | (Intercept) | -25.79 | 5.90 | 0.00 | 1.00 | 0.00 | *** |
| feeding_LRE | pool_size | -0.01 | 0.37 | 0.00 | 1.00 | 0.97 |  |

**Table S5**. Results of the SEM for all bats (overall) and for each of the three bat feeding guilds. Crit. value, critical value (estimate divided by SE); df, degrees of freedom; estimate, estimated value of predictor effects; p‐value, indicates the significance of the predictor effects: *p < 0.05, **p < 0.01, ***p < 0.001; SE, standard error of the estimate; Std. estimate, standardized estimate indicating the effect size in standardized units.

| **Overall** |  |  |  |  |  |  |  |  |
| --- | --- | --- | --- | --- | --- | --- | --- | --- |
| **Response** | **Predictor** | **Estimate** | **Std.Error** | **DF** | **Crit.Value** | **P.Value** | **Std.Estimate** | |
| Standing deadwood density | Beaver presence | 0.80 | 0.08 | 237.01 | 103.66 | 0.00 | 0.40 | *** |
| Canopy heterogeneity | Beaver presence | 0.51 | 0.09 | 237.02 | 30.72 | 0.00 | 0.26 | *** |
| Arthropod abundance | Standing deadwood density | 0.18 | 0.08 | 236.94 | 5.11 | 0.02 | 0.18 | * |
| Arthropod abundance | Canopy heterogeneity | 0.04 | 0.07 | 241.18 | 0.36 | 0.55 | 0.04 |  |
| Arthropod abundance | Beaver presence | 0.13 | 0.12 | 238.74 | 1.18 | 0.28 | 0.06 |  |
| Richness | Arthropod abundance | 0.04 | 0.07 | 220.78 | 0.23 | 0.64 | 0.04 |  |
| Richness | Standing deadwood density | 0.25 | 0.09 | 159.71 | 7.05 | 0.01 | 0.25 | ** |
| Richness | Canopy heterogeneity | -0.07 | 0.08 | 202.98 | 0.79 | 0.38 | -0.07 |  |
| Richness | Beaver presence | 0.31 | 0.14 | 240.64 | 5.04 | 0.03 | 0.16 | * |
| Feeding | Richness | 0.11 | 0.05 | 235.92 | 5.92 | 0.02 | 0.11 | * |
| Feeding | Standing deadwood density | -0.14 | 0.07 | 239.16 | 3.63 | 0.06 | -0.14 |  |
| Feeding | Canopy heterogeneity | 0.04 | 0.06 | 239.85 | 0.54 | 0.46 | 0.04 |  |
| Feeding | Arthropod abundance | 0.14 | 0.05 | 238.96 | 6.09 | 0.01 | 0.14 | * |
| Feeding | Beaver presence | 0.45 | 0.10 | 235.04 | 19.91 | 0.00 | 0.23 | *** |
| Activity | Richness | 0.30 | 0.04 | 232.42 | 64.84 | 0.00 | 0.30 | *** |
| Activity | Feeding | 0.68 | 0.05 | 89.02 | 195.33 | 0.00 | 0.68 | *** |
| Activity | Standing deadwood density | 0.10 | 0.05 | 66.05 | 3.51 | 0.07 | 0.10 |  |
| Activity | Canopy heterogeneity | 0.07 | 0.04 | 114.10 | 2.85 | 0.09 | 0.07 |  |
| Activity | Arthropod abundance | -0.02 | 0.04 | 138.51 | 0.25 | 0.61 | -0.02 |  |
| Activity | Beaver presence | -0.03 | 0.08 | 238.72 | 0.11 | 0.74 | -0.01 |  |
|  |  |  |  |  |  |  |  |  |
| **Short-range Echolocation Guild (SRE)** | |  |  |  |  |  |  |  |
| **Response** | **Predictor** | **Estimate** | **Std.Error** | **DFh** | **Crit.Value** | **P.Value** | **Std.Estimate** | |
| Standing deadwood density | Beaver presence | 0.80 | 0.08 | 237.01 | 103.66 | 0.00 | 0.40 | *** |
| Canopy heterogeneity | Beaver presence | 0.51 | 0.09 | 237.02 | 30.72 | 0.00 | 0.26 | *** |
| Arthropod abundance | Standing deadwood density | 0.18 | 0.08 | 236.94 | 5.11 | 0.02 | 0.18 | * |
| Arthropod abundance | Canopy heterogeneity | 0.04 | 0.07 | 241.18 | 0.36 | 0.55 | 0.04 |  |
| Arthropod abundance | Beaver presence | 0.13 | 0.12 | 238.74 | 1.18 | 0.28 | 0.06 |  |
| Richness | Arthropod abundance | 0.07 | 0.07 | 213.26 | 0.89 | 0.35 | 0.07 |  |
| Richness | Standing deadwood density | 0.18 | 0.09 | 145.28 | 4.06 | 0.05 | 0.19 | * |
| Richness | Canopy heterogeneity | 0.03 | 0.07 | 192.43 | 0.16 | 0.69 | 0.03 |  |
| Richness | Beaver presence | -0.26 | 0.13 | 240.87 | 3.94 | 0.05 | -0.14 | * |
| Feeding | Richness | 0.05 | 0.03 | 236.19 | 3.66 | 0.06 | 0.10 |  |
| Feeding | Standing deadwood density | -0.14 | 0.04 | 236.53 | 12.50 | 0.00 | -0.29 | *** |
| Feeding | Canopy heterogeneity | 0.10 | 0.03 | 239.84 | 8.68 | 0.00 | 0.20 | ** |
| Feeding | Arthropod abundance | 0.06 | 0.03 | 239.92 | 4.40 | 0.04 | 0.13 | * |
| Feeding | Beaver presence | 0.19 | 0.06 | 236.11 | 11.00 | 0.00 | 0.19 | ** |
| Activity | Richness | 0.19 | 0.02 | 235.02 | 69.67 | 0.00 | 0.26 | *** |
| Activity | Feeding | 0.88 | 0.05 | 238.99 | 259.23 | 0.00 | 0.61 | *** |
| Activity | Standing deadwood density | 0.01 | 0.03 | 235.62 | 0.03 | 0.86 | 0.01 |  |
| Activity | Canopy heterogeneity | 0.11 | 0.03 | 238.15 | 15.05 | 0.00 | 0.16 | *** |
| Activity | Arthropod abundance | 0.08 | 0.03 | 238.91 | 9.60 | 0.00 | 0.12 | ** |
| Activity | Beaver presence | -0.03 | 0.05 | 234.89 | 0.40 | 0.53 | -0.02 |  |
| **Mid-range Echolocation Guild (MRE)** | |  |  |  |  |  |  |  |
| **Response** | **Predictor** | **Estimate** | **Std.Error** | **DF** | **Crit.Value** | **P.Value** | **Std.Estimate** | |
| Standing deadwood density | Beaver presence | 0.80 | 0.08 | 237.01 | 103.66 | 0.00 | 0.40 | *** |
| Canopy heterogeneity | Beaver presence | 0.51 | 0.09 | 237.02 | 30.72 | 0.00 | 0.26 | *** |
| Arthropod abundance | Standing deadwood density | 0.18 | 0.08 | 236.94 | 5.11 | 0.02 | 0.18 | * |
| Arthropod abundance | Canopy heterogeneity | 0.04 | 0.07 | 241.18 | 0.36 | 0.55 | 0.04 |  |
| Arthropod abundance | Beaver presence | 0.13 | 0.12 | 238.74 | 1.18 | 0.28 | 0.06 |  |
| Richness | Arthropod abundance | 0.00 | 0.05 | 239.63 | 0.01 | 0.93 | 0.01 |  |
| Richness | Standing deadwood density | 0.25 | 0.07 | 220.02 | 12.91 | 0.00 | 0.31 | *** |
| Richness | Canopy heterogeneity | -0.02 | 0.06 | 235.57 | 0.18 | 0.68 | -0.03 |  |
| Richness | Beaver presence | 0.38 | 0.10 | 238.62 | 14.80 | 0.00 | 0.24 | *** |
| Feeding | Richness | 0.48 | 0.11 | 239.58 | 19.05 | 0.00 | 0.24 | *** |
| Feeding | Standing deadwood density | -0.19 | 0.12 | 225.15 | 2.44 | 0.12 | -0.12 |  |
| Feeding | Canopy heterogeneity | -0.03 | 0.10 | 238.73 | 0.11 | 0.74 | -0.02 |  |
| Feeding | Arthropod abundance | 0.18 | 0.09 | 239.97 | 4.04 | 0.05 | 0.12 | * |
| Feeding | Beaver presence | 0.54 | 0.17 | 235.24 | 9.89 | 0.00 | 0.17 | ** |
| Activity | Richness | 0.49 | 0.07 | 231.20 | 43.23 | 0.00 | 0.29 | *** |
| Activity | Feeding | 0.49 | 0.04 | 174.42 | 133.01 | 0.00 | 0.58 | *** |
| Activity | Standing deadwood density | 0.10 | 0.07 | 75.15 | 1.91 | 0.17 | 0.08 |  |
| Activity | Canopy heterogeneity | 0.00 | 0.06 | 142.13 | 0.00 | 0.96 | 0.00 |  |
| Activity | Arthropod abundance | -0.07 | 0.06 | 166.20 | 1.40 | 0.24 | -0.05 |  |
| Activity | Beaver presence | 0.11 | 0.12 | 238.56 | 0.84 | 0.36 | 0.04 |  |
|  |  |  |  |  |  |  |  |  |
| **Long-range Echolocation Guild (LRE)** | | |  |  |  |  |  |  |
| **Response** | **Predictor** | **Estimate** | **Std.Error** | **DF** | **Crit.Value** | **P.Value** | **Std.Estimate** | |
| Standing deadwood density | Beaver presence | 0.80 | 0.08 | 237.01 | 103.66 | 0.00 | 0.40 | *** |
| Canopy heterogeneity | Beaver presence | 0.51 | 0.09 | 237.02 | 30.72 | 0.00 | 0.26 | *** |
| Arthropod abundance | Standing deadwood density | 0.18 | 0.08 | 236.94 | 5.11 | 0.02 | 0.18 | * |
| Arthropod abundance | Canopy heterogeneity | 0.04 | 0.07 | 241.18 | 0.36 | 0.55 | 0.04 |  |
| Arthropod abundance | Beaver presence | 0.13 | 0.12 | 238.74 | 1.18 | 0.28 | 0.06 |  |
| Richness | Arthropod abundance | -0.02 | 0.06 | 231.83 | 0.17 | 0.68 | -0.03 |  |
| Richness | Standing deadwood density | 0.03 | 0.07 | 187.73 | 0.16 | 0.69 | 0.04 |  |
| Richness | Canopy heterogeneity | -0.09 | 0.06 | 220.34 | 2.06 | 0.15 | -0.11 |  |
| Richness | Beaver presence | 0.36 | 0.10 | 239.96 | 12.36 | 0.00 | 0.24 | *** |
| Activity | Richness | 0.10 | 0.01 | 239.62 | 153.85 | 0.00 | 0.57 | *** |
| Activity | Standing deadwood density | 0.01 | 0.01 | 209.05 | 2.83 | 0.09 | 0.11 |  |
| Activity | Canopy heterogeneity | -0.02 | 0.01 | 229.77 | 5.36 | 0.02 | -0.13 | * |
| Activity | Arthropod abundance | -0.02 | 0.01 | 236.57 | 6.59 | 0.01 | -0.13 | * |
| Activity | Beaver presence | -0.03 | 0.01 | 237.98 | 4.05 | 0.05 | -0.10 | * |

**
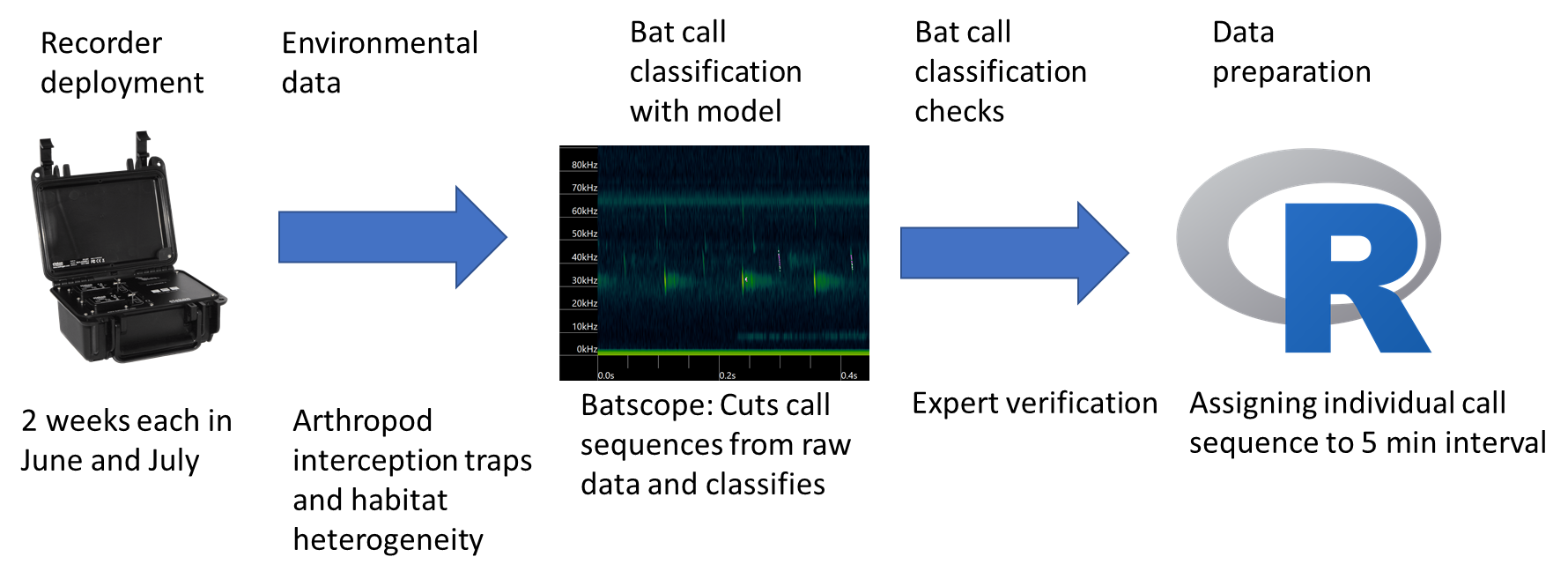
**

**Figure S1.** Schematic protocol for analysing the bat acoustic data. Autonomous bat recorders were deployed for two weeks. Following data collection, the raw data files were processed with Batscope (Obrist et al. 2018), a program that cuts and classifies bat calls based on machine learning. Experts verified the results. Further data preparation was conducted in R (R Core Team 2024) by assigning call sequences into 5-minute intervals. Sources: Batlogger with permission from batlogger.com, R-Logo from https://www.r-project.org/logo/Rlogo.svg under CC-BY-SA 4.0 license.


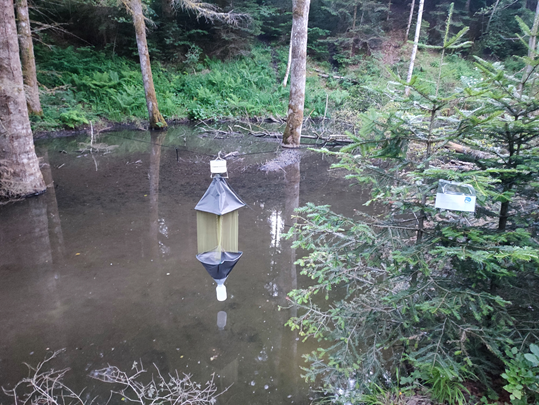

**Figure S2**. Flight interception trap installed over the pool behind the beaver dam in one of the stream ecosystems included in our study. Photo credits: Valentin Moser


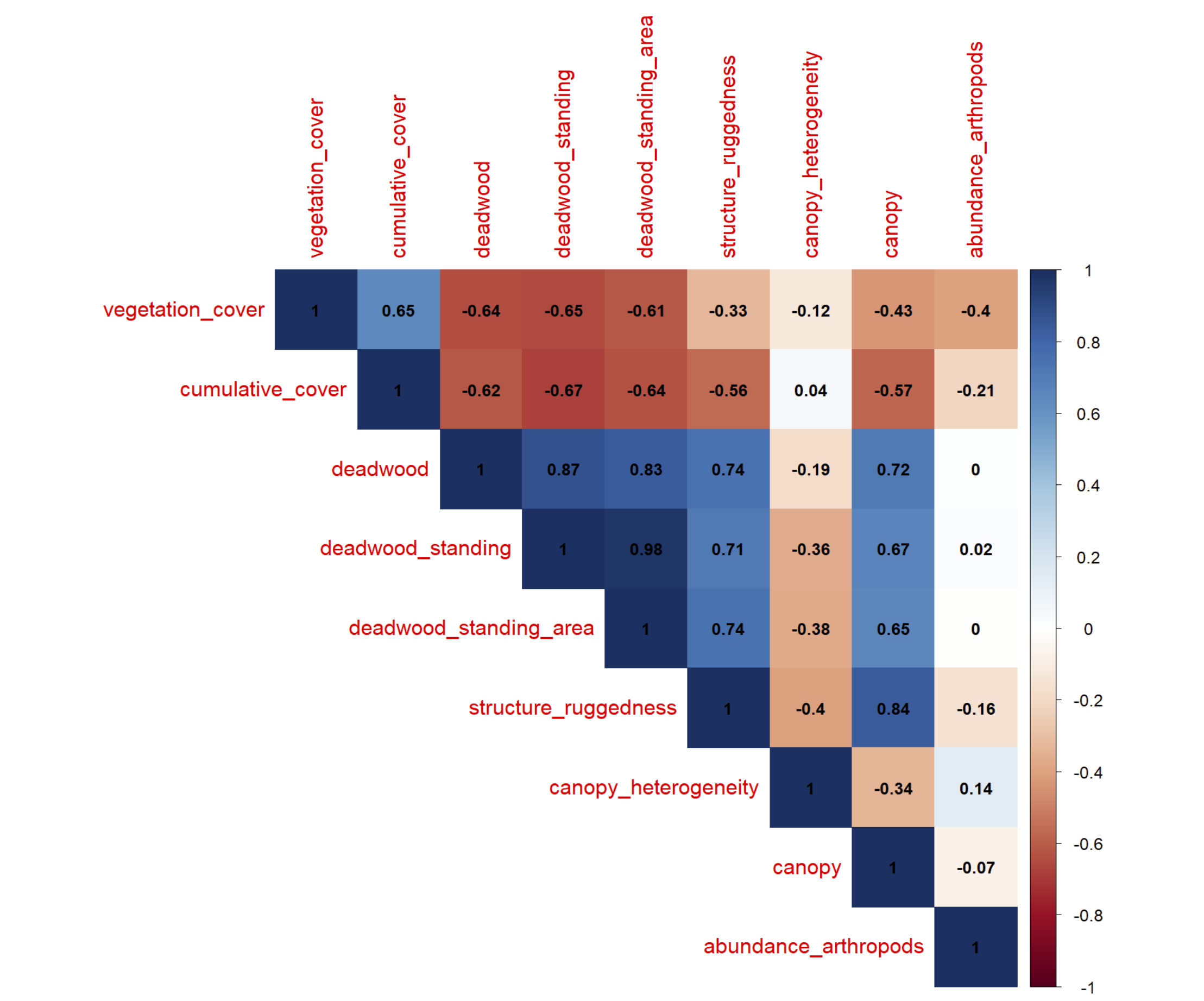


**Figure S3.** Spearman correlations between the habitat variables and abundance of arthropods included in our study. abundance_arthropods = abundance arthropods; canopy = canopy cover (proportion between 0 and 1); canopy_heterogeneity = canopy heterogeneity (unitless modelled value); cumulative_cover = cumulative cover of herb, shrub and tree layer (%); deadwood = deadwood (total deadwood volume in m3); deadwood_standing = total standing deadwood (standing deadwood volume in m3); deadwood_standing_area = standing deadwood density (standing deadwood volume in m3 divided by survey area in m2); structure_ruggedness = structure ruggedness (unitless modelled value); vegetation_cover = vegetation cover (%).


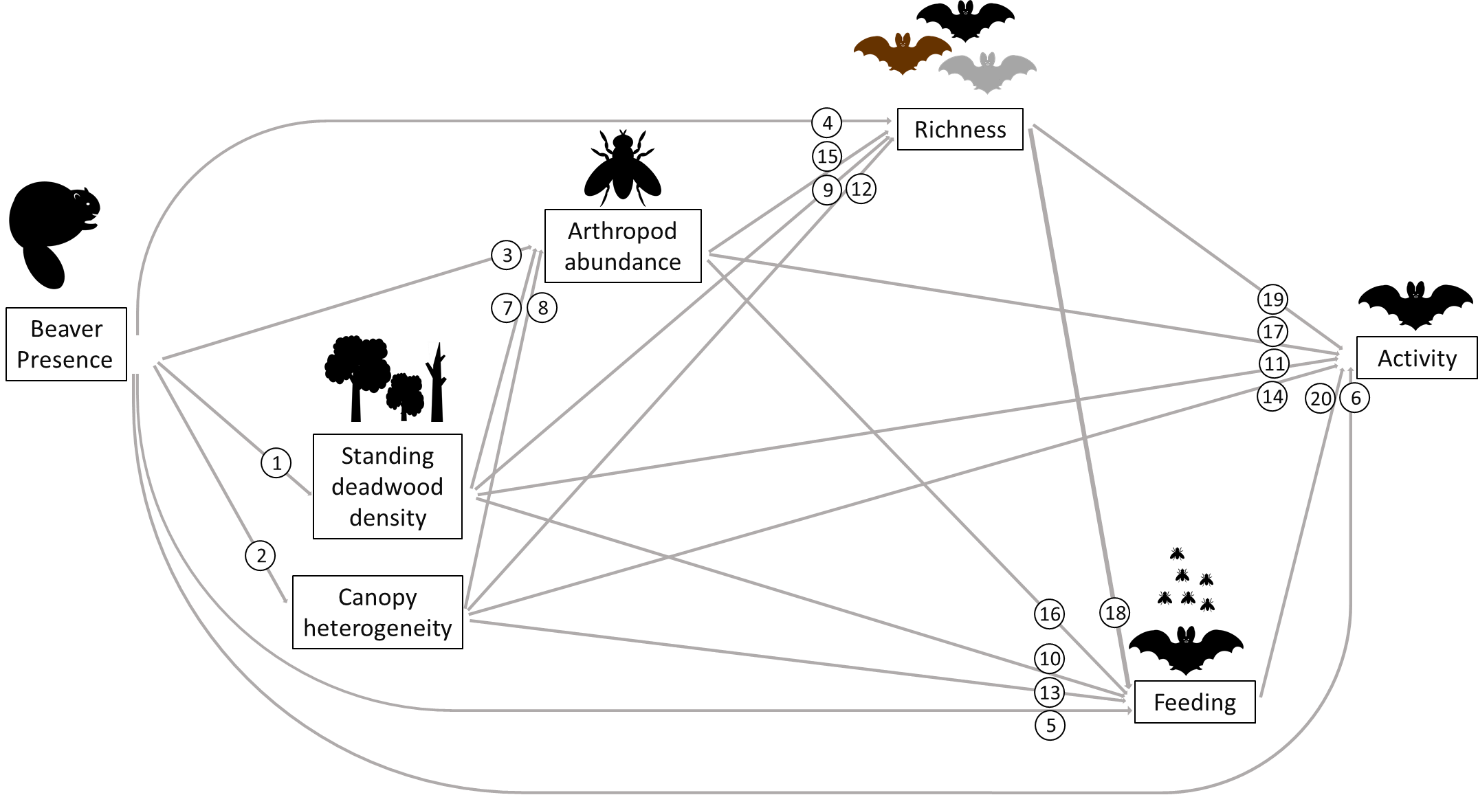


**Figure S4**. A priori SEM model. The arrows represent all possible pathways based on our hypothesis. The path numbers correspond to numbers in Table 1, which indicates probable mechanisms and references. All paths are presumed to be positive and going from left to right.

**Figure S5**. Standardized direct (light blue), indirect (blue) and total effects (dark blue) of all variables on activity per bat feeding guild, calculated from the Structural Equation Models.


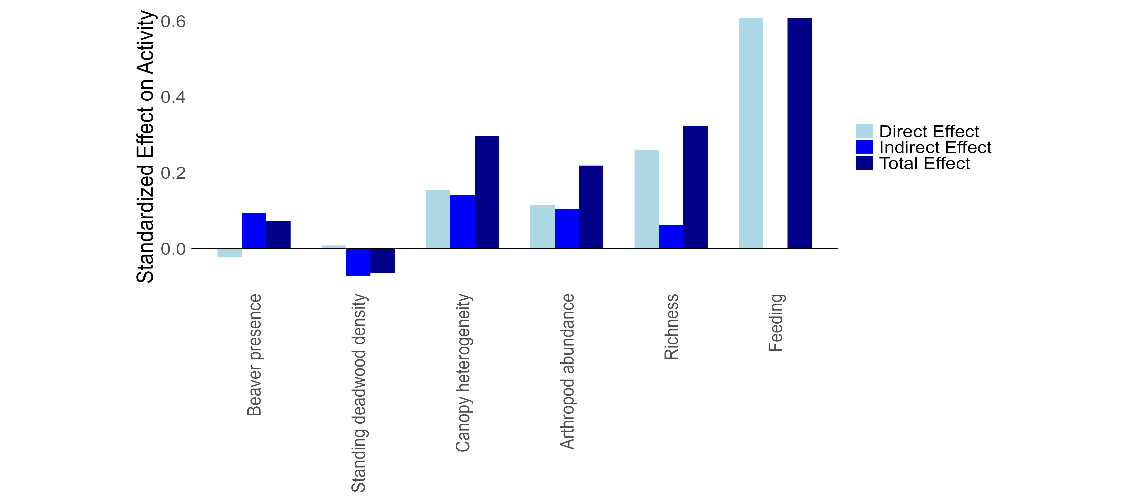

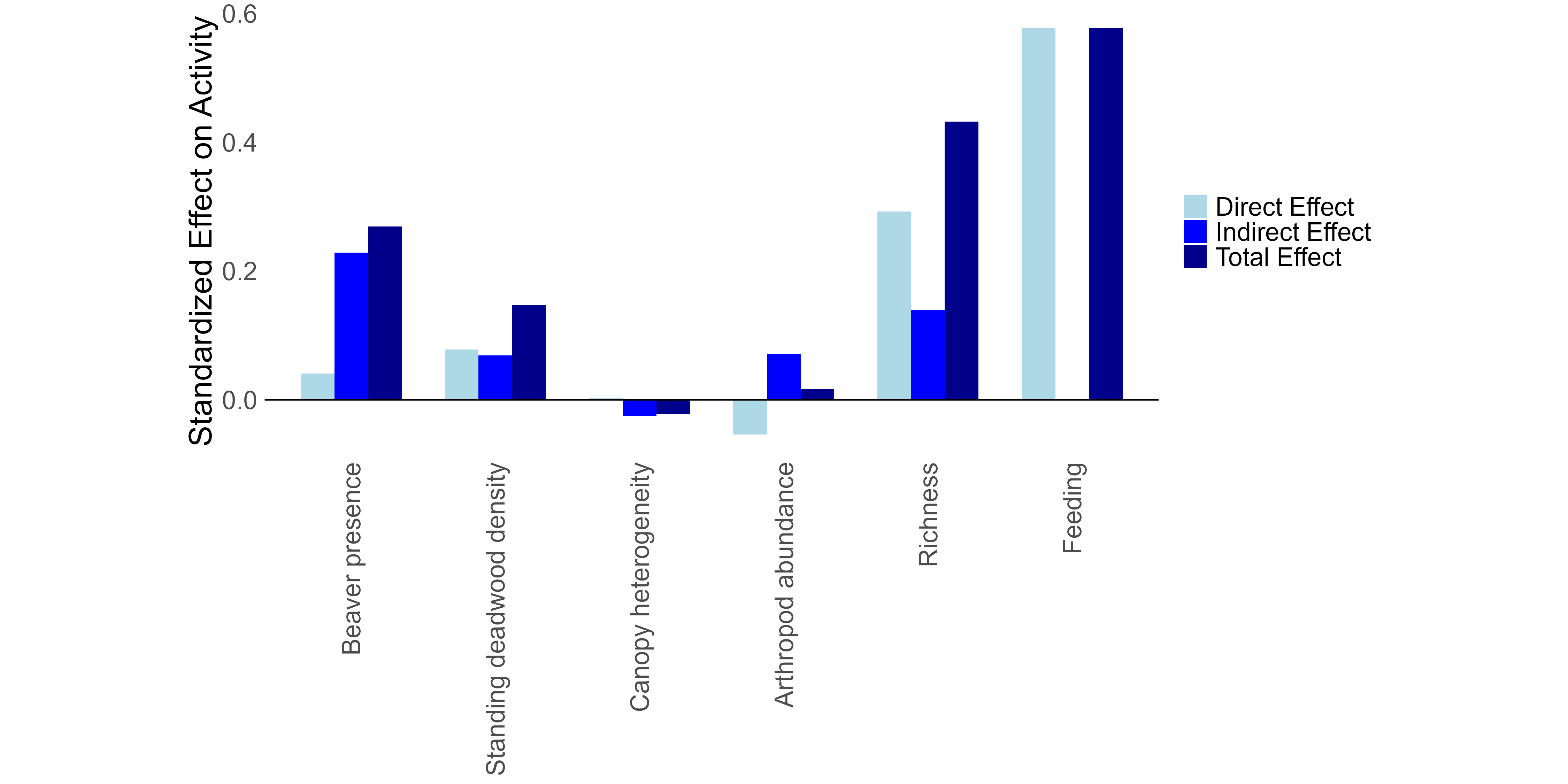

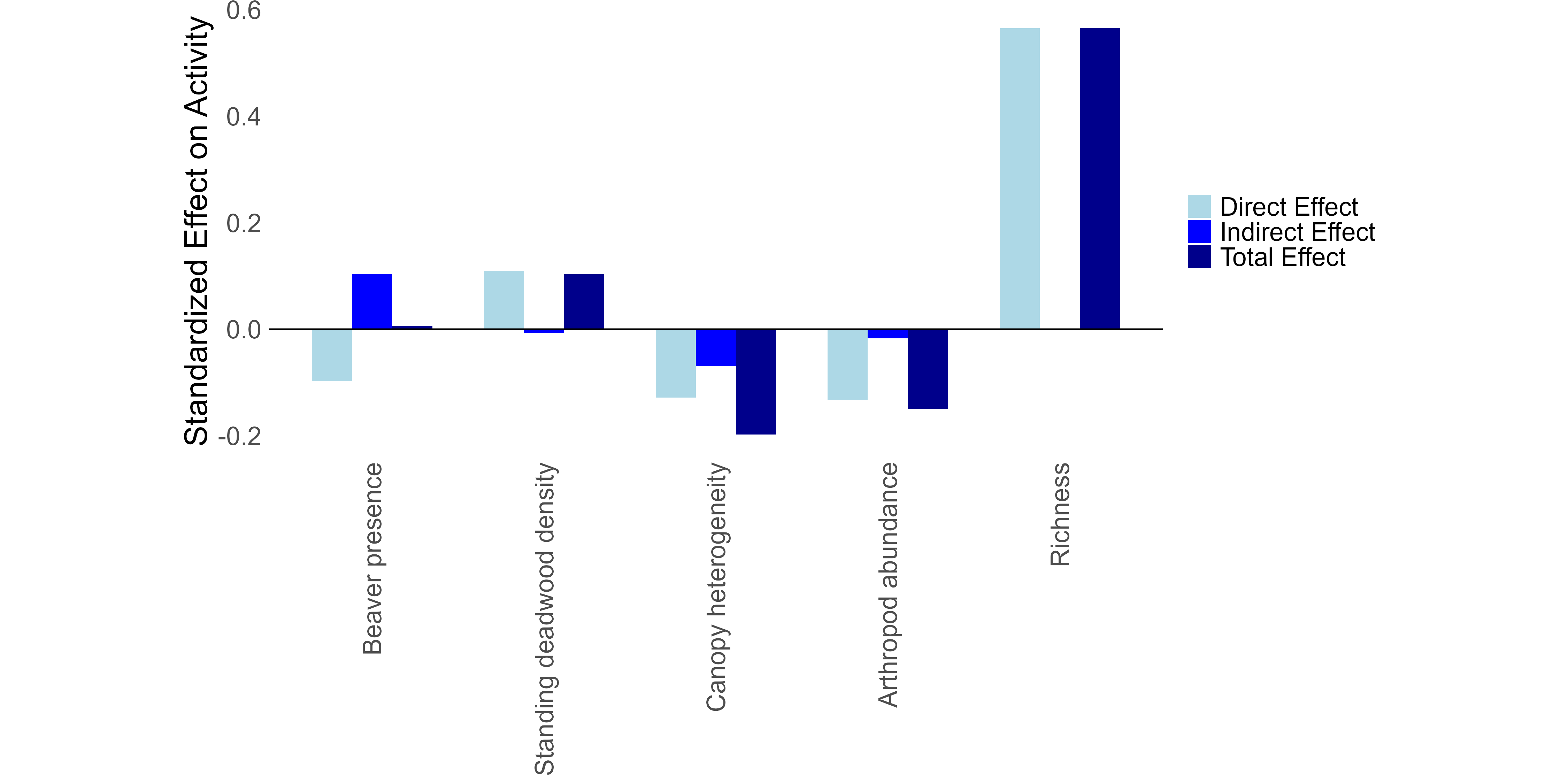


**Mid-Range Echolocation Guild (MRE)**

**Short-Range Echolocation Guild (SRE)**

**Long-Range Echolocation Guild (LRE)**
